# Natural products and their semi-synthetic derivatives against antimicrobial-resistant human pathogenic bacteria and fungi

**DOI:** 10.1016/j.sjbs.2022.103376

**Published:** 2022-07-18

**Authors:** Hafsa Qadri, Abdul Haseeb Shah, Syed Mudasir Ahmad, Bader Alshehri, Abdullah Almilaibary, Manzoor Ahmad Mir

**Affiliations:** a Department of Bioresources, School of Biological Sciences, University of Kashmir, Srinagar 190006, India; b Division of Animal Biotechnology, SKUAST-K, Shuhama-190006, J&K, India; c Department of Medical Laboratory Science, College of Applied Medical Sciences, Majmaah University, Majmaah 11952, Saudi Arabia; d Department of Family & Community Medicine, Faculty of Medicine, Albaha University, Alaqiq 307501, Saudi Arabia

**Keywords:** Microbial diseases/infections, Antimicrobial drug resistance (AMR), Antimicrobial agents, Pathogenic microbes, Natural products, **AMR**, Antimicrobial resistance, **AMPs**, Antimicrobial peptides, **ICMR**, Indian Council of Medical Research, **MDR**, Multidrug resistance, **ICU**, Intensive Care Unit, WHO, World Health Organization, **USFDA**, US Food and Drug Administration, **NMPA**, National Medical Products Administration

## Abstract

Human infectious diseases caused by various microbial pathogens, in general, impact a large population of individuals every year. These microbial diseases that spread quickly remain to be a big issue in various health-related domains and to withstand the negative drug impacts, the antimicrobial-resistant pathogenic microbial organisms (pathogenic bacteria and pathogenic fungi) have developed a variety of resistance processes against many antimicrobial drug classes. During the COVID-19 outbreak, there seems to be an upsurge in drug and multidrug resistant-associated pathogenic microbial species. The preponderance of existing antimicrobials isn’t completely effective, which limits their application in clinical settings. Several naturally occurring chemicals produced from bacteria, plants, animals, marine species, and other sources are now being studied for antimicrobial characteristics. These natural antimicrobial compounds extracted from different sources have been demonstrated to be effective against a variety of diseases, although plants remain the most abundant source. These compounds have shown promise in reducing the microbial diseases linked to the development of drug tolerance and resistance. This paper offers a detailed review of some of the most vital and promising natural compounds and their derivatives against various human infectious microbial organisms. The inhibitory action of different natural antimicrobial compounds, and their possible mechanism of antimicrobial action against a range of pathogenic fungal and bacterial organisms, is provided. The review will be useful in refining current antimicrobial (antifungal and antibacterial) medicines as well as establishing new treatment strategies to tackle the rising number of human bacterial and fungal-associated infections.

## Highlights

• COVID-19 pandemic has traumatized the entire world. During this outbreak, an upsurge in MDR-associated pathogenic microbial organisms has been recorded.
• The increasing human microbial diseases pose a severe danger to global human safety.
• The infectious microbes have developed multiple tolerance strategies to overcome the negative drug impacts.
• Several naturally occurring chemicals produced from bacteria, plants, animals, marine species, and other sources with antimicrobial characteristics have been reviewed.
• These compounds show promise in minimizing the globally increasing microbial diseases.

## Graphical abstract

**Figure:**
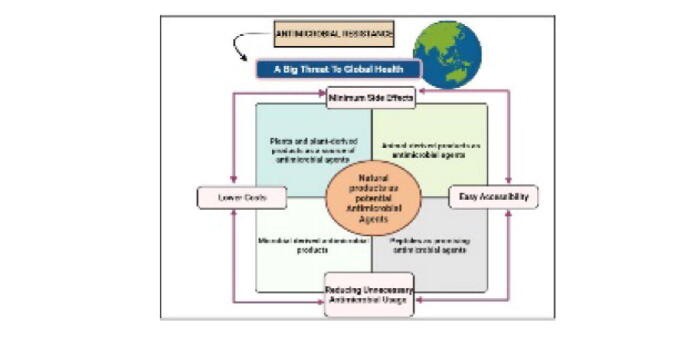

## 1 Introduction

Antimicrobial resistance (AMR) poses a severe population health danger to advances made in infectious disease treatment, cancer treatment, organ transplantation, and critical care (O'Neill 2016). This developing issue is as heavy as many other global issues like climate change, and it necessitates effective management and response (Ayukekbong et al., 2017). Drug-resistant diseases caused by AMR kill roughly 700,000 people worldwide each year, and without effective intervention, 10 million individuals are expected to expire and the global economy will lose around a hundred trillion dollars around the year 2050 (O'Neill 2016). In South Asia, India has experienced one of the largest increases in age-standardized infectious disease mortality, and the phenomenon of antibiotic resistance is on the rise. Meanwhile, India is the world leader in human antibiotic usage, which represents a notable factor in AMR. The use of a spectrum of pragmatic antibiotic therapies increases as resistance spreads, limiting potential treatments and affecting outcomes for patients. Excessive antibiotic utilization, insufficient knowledge, improper utilization of diagnostic procedures, overpopulation, cross-infections, poor health infrastructural facilities, etc. as well intensify the issue of AMR in India (Manesh and Varghese 2021). The Antimicrobial Resistance Surveillance Research Network was developed by the Indian Council of Medical Research (ICMR) to assess the impact of resistance amongst seven important pathogenic organisms and undertake stewardship actions (Walia et al., 2019). Antimicrobial tolerance represents a worldwide concern as a result of the widening range of various drug resistance processes (Brown and Wright 2016). The rapidly rising AMR problem must be addressed right away. Antimicrobial drugs as therapy choices for the developing multidrug-resistant (MDR) microbial diseases are limited, and there are a variety of negative factors contributing to the emergence of such a growing AMR epidemic (Qadri et al., 2021). India has been a member of the Global Antimicrobial Resistance Surveillance System since the year 2017. Even though surveillance is in place, the clinical presentation of drug-resistant infections is still unclear, leading to a loop of incorrect medication and tolerance strengthening (Manesh and Varghese 2021).

**Fig. 1:**
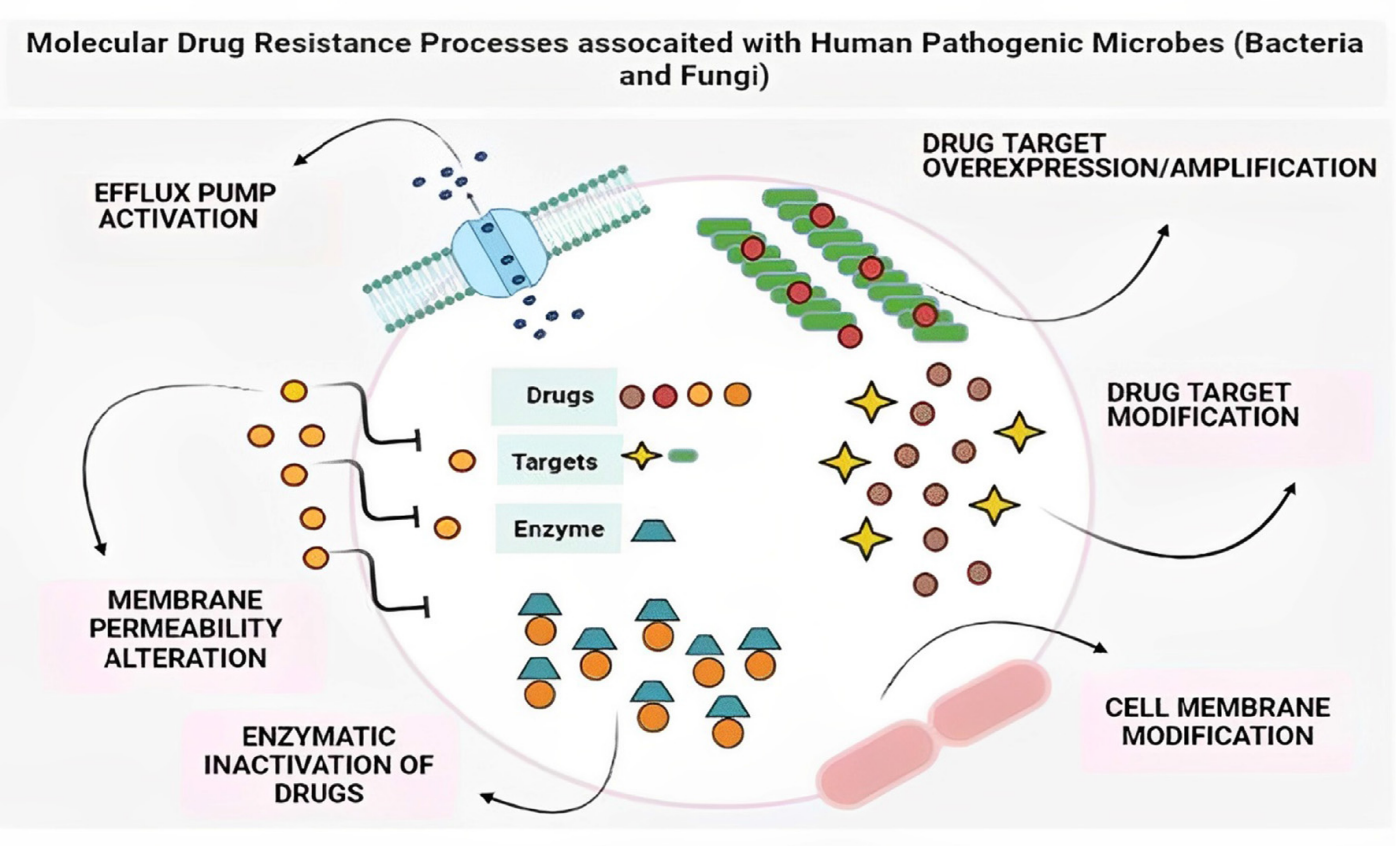
Modifications and up-regulation/activation of drug efflux pumps, expansion of the drug-target molecule, and other molecular drug resistance procedures used by diverse pathogenic microbial species (pathogenic bacteria and fungi) to counter the impacts of multiple antimicrobial drugs are represented in this figure.

AMR is a major public health issue around the world (Prestinaci et al., 2015). Excess microbial exposure to antibiotic compounds, primarily due to their abuse in agricultural and healthcare centers, is one of the major drivers of the AMR phenomenon (Capozzi et al., 2013).Overuse/misuse of antibiotics, unsuitable and wrongly given antibiotics, large agricultural utilization of antibiotics, and the accessibility of only a few innovative antibiotics are the factors contributing to the establishment of antibiotic tolerance issues and crises (Ventola 2015). On the other hand, due to scientific constraints, clinical impediments, and poor economic yields, development in discovering novel antimicrobial agents has slowed (Payne et al., 2015). Since the quality of pharmaceutical drugs is still in question, developing countries and regions with low resources, a defective pharmaceutical supply chain, and poor health service management systems may contribute most to the development of the AMR phenomenon (Chokshi et al., 2019). AMR propagation has been accelerated during and after the COVID-19 outbreak due to overutilization and misuse of current antimicrobial drugs, in addition to other well-known variables that drive the AMR phenomenon (Lobie et al., 2021). AMR is a complex issue that must be tackled in the same way as other global issues such as climate change and global warming (Ayukekbong et al., 2017). Hence, there is an urgent requirement for the establishment of novel tactics and ways to address the issue of growing drug tolerance associated with different pathogenic bacterial and fungal organisms. Different molecular, genetic, and immunological processes must be established to effectively manage the expanding number of infections in humans. (Mir, 2015, Mir and Agrewala, 2007, Mir and Al-baradie, 2013, Mir and Albaradie, 2014, Sheikh et al., 2021, Mir, 2021, Sheikh et al., 2021). To cope with the negative drug impacts, these pathogenic microorganisms have developed different tolerance processes (Fig. 1) against many antimicrobial classes.

**Fig. 2:**
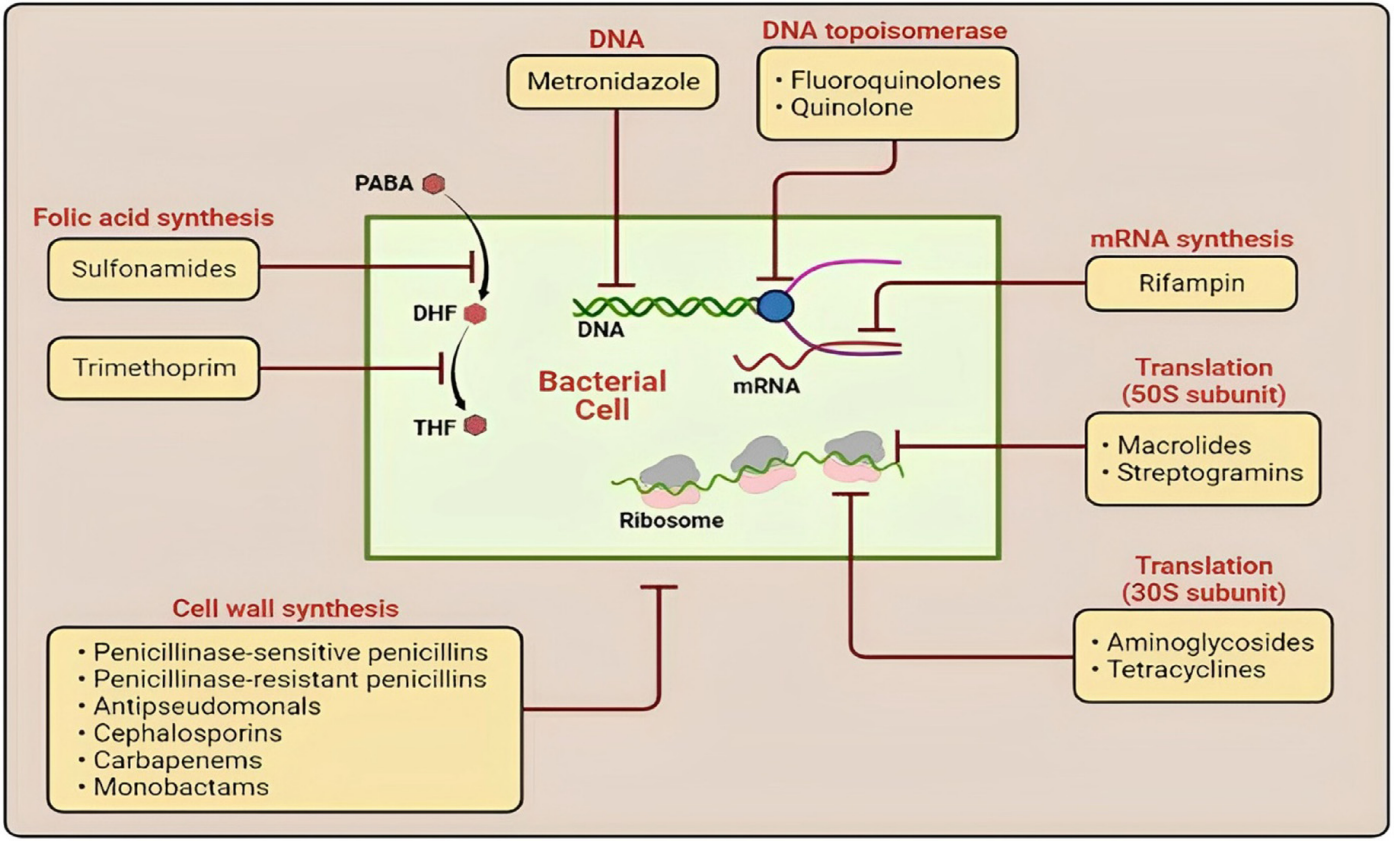
Diagrammatic representation of the most utilized classes of antibacterial agents (Monobactams, Aminoglycosides, Macrolides, Cephalosporins, Rifampin, Sulfonamides, etc.) with major sites of action (Cell wall, plasma membrane, ribosomes, etc.).

**Fig. 3:**
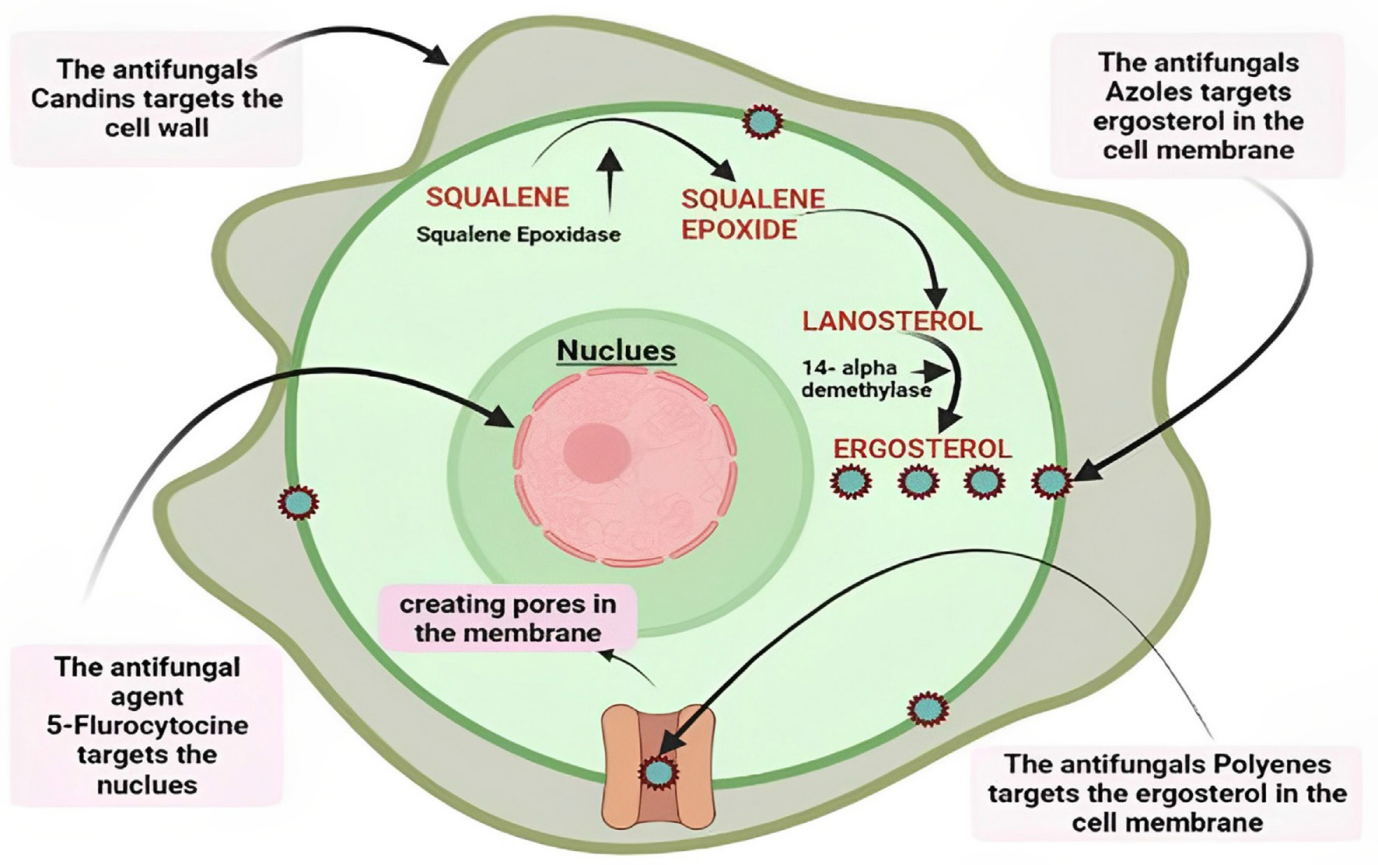
Diagrammatic representation of the most utilized antifungal drugs with major sites of action. In general, antifungal drugs like Candins attack the fungal cell wall, destroying it and allowing other antifungal agents to work. Furthermore, antifungals such as polyenes and azoles target ergosterol, an important cell membrane ingredient, inducing cell death and allowing 5-flourocytosine to target nuclei and disrupt DNA/RNA synthesis.

Antimicrobials (antibacterial and antifungal) of different classes are being continuously utilized **(**Fig. 2 **and** Fig. 3**)** (Qadri et al., 2021). The majority of existing antimicrobials aren't completely effective, which limits their application in clinical practice. Even though synthetic antimicrobial drugs have indeed been approved throughout many countries, several researchers are interested in the use of natural substances obtained from different organisms viz: microbes, plants, and animals (Gyawali and Ibrahim, 2014, Moloney, 2016). These substances have shown the potential in preventing infections associated with increased drug tolerance (Rossiter et al., 2017). We present a comprehensive summary of the most important natural products and their derivatives against different human pathogenic bacterial and fungal organisms in this review. The review will be valuable in giving information for improving the current antimicrobial (antifungal and antibacterial) therapies and developing novel treatment techniques to combat the rising number of bacterial and fungal infections.

### 1.1 COVID-19 pandemic and the increasing antimicrobial resistance

As per many recent reports, it has been found that during the COVID-19 outbreak, there was an upsurge in MDR-associated pathogenic microbial organisms (Contou et al., 2020, Li et al., 2020, Posteraro et al., 2020, Mohamed et al., 2021). SARS-CoV-2 (severe acute respiratory syndrome coronavirus 2) infection is minor in most people, but co-infection can enhance a patient's vulnerability to serious illnesses by weakening the system of defense (Netea et al., 2020). The cause for increasing multidrug-resistant pathogenic microbial organisms is multifactorial and especially associated with increased rates of vigorous antimicrobial usage in COVID-19 individuals having a minimum risk of secondary or co-infection (Lai et al., 2021). According to a recent study, COVID-19 patients had significantly decreased gut bacterial diversity, opportunistic pathogens like Streptococcus, *Veillonella, Actinomyces*, etc. are significantly higher, and beneficial symbionts such as *Blautia, Collinsella*, etc are significantly reduced (Gu et al., 2020). However, another study in a descriptive investigation found that the co-infected fungal organisms comprised *Aspergillus spp*., *Candida albicans,* and *Candida glabrata* (Chen et al., 2020). A study done by Li et al. in Wuhan, China, found that *Acinetobacter baumannii* was the most prevalent infectious organism among 159 bacterial strains collected from 102 COVID-19 patients (having acquired secondary bacterial diseases), following *Klebsiella pneumoniae,* etc (Li et al., 2020). Another retrospective analysis conducted in a French ICU (Intensive Care Unit) discovered that 26 COVID-19 patients admitted to the ICU for acute respiratory failure had been co-infected with a pathogenic bacterial strain, 2 of which were tolerant to 3rd-generation cephalosporins and 5 of which were tolerant to the antibiotic agent amoxicillin/clavulanate (Contou et al., 2020). According to a study report, 5 COVID-19 patients from New York were co-infected with New Delhi Metallo-lactamase (NDM)-producing *Enterobacter cloacae*. Co-infections with fungi in COVID-19 victims are a frequent clinical challenge. According to a report, a COVID-19 patient having type 2 diabetes mellitus suffered three bouts of secondary bloodstream infection caused by the human pathogenic organism *Candida glabrata* and others (Posteraro et al., 2020). Another study reported that an Irish patient having acute COVID-19 pneumonia was worsened by a lethal co-infection with a multi-triazole-tolerant *Aspergillus fumigatus* strain (Mohamed et al., 2021). Antibiotic stewardship concepts such as quality assessment and proactive infection control strategies, as well as adequate medication and improved antimicrobial administration, could reduce the emergence of such pathogens during this outbreak (Lai et al., 2021).

Many research studies have reported different mechanisms associated with COVID-19 effects which contribute to the development of the growing AMR phenomenon (Lobie et al., 2021). Previous research has documented how bacteria developed tolerance to alcohol-based sanitizers via unknown genetic and molecular processes (Pidot et al., 2018). Sanitizer ingredients like alcohol, phenols, surfactants, etc. which induce microbial DNA damage, or benzalkonium chloride (BAC) and others, with multiple antimicrobial characteristics, could develop this resistance (Tagkopoulos 2019). Almost all cleaning and hand sanitizing products, both homemade and retail, contain harmful agents like hydrogen peroxide, etc that cause microbial DNA damage (da Silva 2016). The stimulation of translesion synthesis polymerases (TLS) by bacteria in response to DNA damage tolerates and bypasses unrepaired DNA lesions, resulting in mutations that lead to the establishment of AMR (Choi et al., 2017). When bacterial organisms are exposed to antibiotic-based disinfectants, they establish a subpopulation that survives and can become antibiotic-resistant. This “selected” subset performs a critical role in biofilm infection recalcitrance (Dorr et al., 2010, Lobie et al., 2021). As a result, microorganisms can change their phenotypic and genotypic characteristics, affecting antibiotic targets (Lewis, 2007, Lobie et al., 2021).

### 1.2 Rising scenario of globally spreading microbial infections

**Fig. 4:**
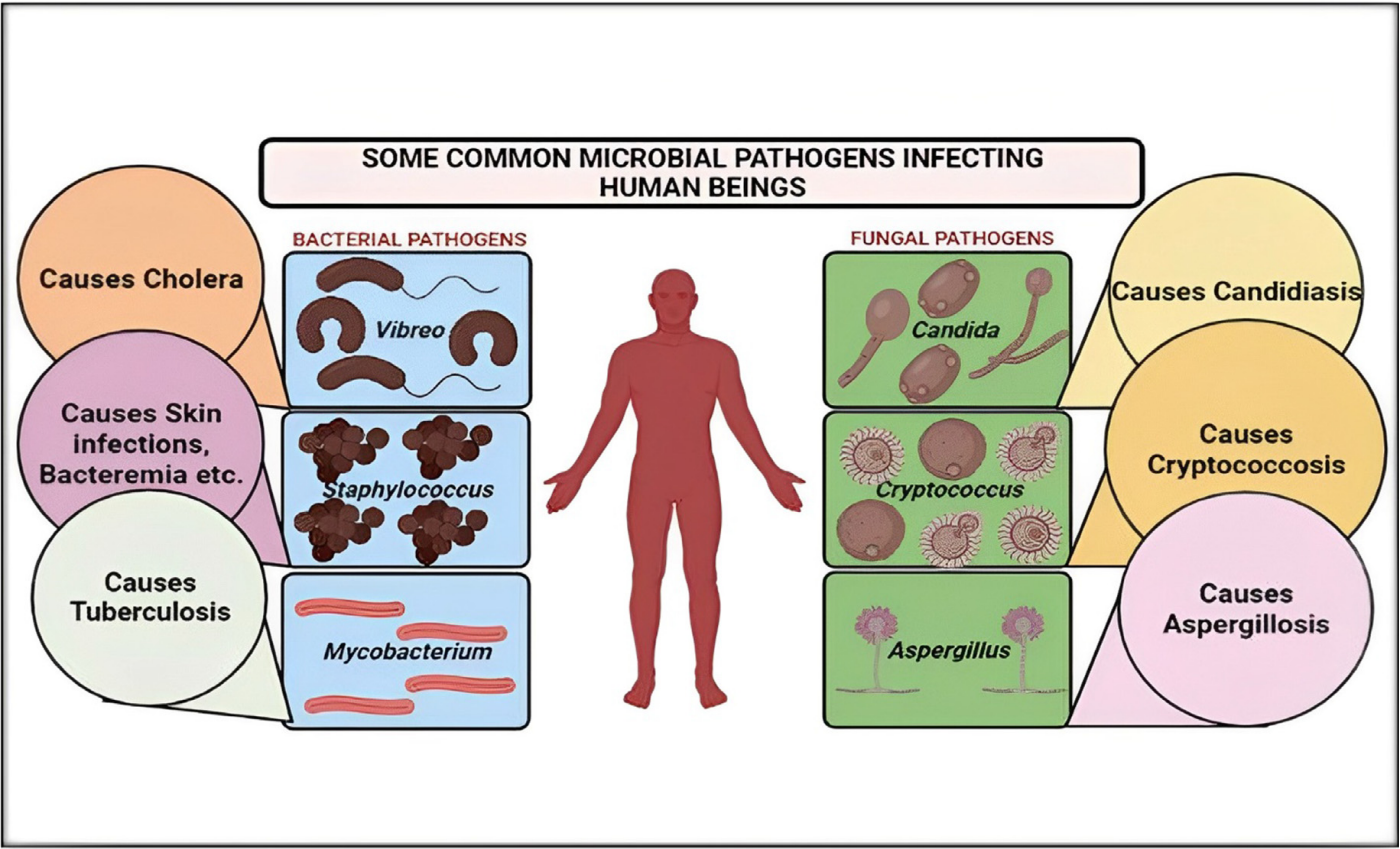
Some of the most prevalent bacterial (*Vibrio*, *Staphylococcus*, *Mycobacterium*) and fungal pathogens (*Candida*, *Aspergillus*, *Cryptococcus*) that infect humans causing various microbial infections are depicted in this diagram.

The phenomenon of Antimicrobial resistance has emerged among the most severe healthcare issues of the 21st century, threatening the successful treatment and therapy of the growing spectrum of microbial diseases associated with bacteria, fungi, etc. which are showing tolerance to the frequently utilized antibiotic agents (Mir et al., 2022, Prestinaci et al., 2015). Microbial organisms like bacteria, fungi, etc. cause different types of infectious diseases. Despite the implementation of many protection/management/control measures, microbial infections remain to be among the world’s major healthcare challenges, accounting for countless deaths each year (Cohen, 2000, Haque et al., 2020). Human microbial infections (Bacterial and Fungal) are increasing at a rapid rate **(**Fig. 4**)**.

Infectious diseases continue to pose a danger to world healthcare systems and economics, necessitating ongoing exploration, investigation, and updating (Cupertino et al., 2020, Mir, 2022, Mir et al., 2022, Mir et al., 2022, Mir et al., 2022, Mir Ahmed Manzoor and Mehraj Umar, 2019). The assessment and establishment of possible molecular/genetic methods for the effective controlling of the worldwide issue of increasing human infectious diseases are required (Mir et al., 2022, Mir et al., 2022, Mir et al., 2022, Mir et al., 2022, Mir et al., 2022, Nicholson, 2016). The World Health Organization has increasingly established the requirement for a more comprehensive and balanced global response to AMR. The WHO Global Strategy for Antimicrobial Resistance Containment was established in the year 2001 to provide a structure of measures to decrease the growth and spread of antimicrobial-resistant pathogenic microbes (Organization, 2001, Prestinaci et al., 2015). The Evolving Threat of Antimicrobial Resistance – Options for Action was released by WHO in the year 2012, and it recommended a combo of initiatives, including improving health and monitoring, upgrading antimicrobial usage in community and hospital, disease prevention and control, promoting the growth of advanced and modern drugs and vaccines, and political support (Organization, 2012, Prestinaci et al., 2015). Following the declaration of surveillance as a major purpose, WHO issued the first global report on AMR surveillance in April in the year 2014, compiling data from national and international surveillance networks. Surveillance information is extremely valuable in guiding options for treatment, detecting AMR patterns, identifying target areas for actions, evaluating the effectiveness of resistance-control measures, etc. The absence of sufficient surveillance in several regions of the world left enormous gaps in our understanding of the phenomenon's spread and scope (Organization, 2014, Prestinaci et al., 2015).

#### 1.2.1 Bacterial infections

Bacterial organisms occur everywhere. They are vital to the preservation of the ecosystem we live in. Diseases/Infections are caused by a small percentage of bacteria and such infections have a big impact on human health. Bacterial infections are often better to handle in comparison to viral infections because of the larger armament of antimicrobial drugs with antibacterial action. The phenomenon of AMR is a growing issue having possibly fatal consequences, even worse than the infections produced by viruses, etc. Bacterial infections/diseases are triggered by various elements. To initiate, an organism's infectivity describes how many people will become infected versus how many will be immune and exposed. Secondly, pathogenicity refers to the ability of an infectious organism/agent to produce disease. Infections are caused by pathogenic microorganisms that have characteristics that enable them to evade the body's defense systems and reap the benefits of their resources. Eventually, virulence refers to an organism's potential to cause disease as evaluated by invasiveness and toxic-chemical generation(Doron and Gorbach 2008).

Pathogenic bacterial organisms adopt multiple antimicrobial resistance mechanisms to overcome the effect of different antibacterial agents (Kapoor et al., 2017). In general AMR mechanisms are divided into 4 major classes: A) drug uptake limitation, B) drug target modification, C) drug inactivation, and D) active drug efflux. Restricted drug uptake, drug inactivation, and drug efflux are some examples of intrinsic resistance mechanisms adopted by different bacterial organisms; while drug target modification, inactivation of the drug, and drug efflux are examples of acquired resistance mechanisms. The different kinds of resistant processes used by gram-negative bacteria and gram-positive bacteria differ due to changes in their structure etc (Reygaert 2018).

#### 1.2.2 Fungal infections

Fungi are widely distributed in the environment, with around 100,000 species, 300 of which can cause infections in the case of both animals and human beings (Gupta et al., 2017). In humans, fungal infections are now difficult to manage. The majority of these fungi infect immune-compromised people who have HIV, asthma, diabetes, undergoing cancer treatments, autoimmune disorder therapies, and other innovative treatments (K. Redhu et al., 2016, Bongomin et al., 2017). *Aspergillus, Cryptococcus,* and *Candida* genera are the most common reason of invasive fungal infections, accounting for over 90% of fungal-related mortality (Bassetti et al., 2016, Ksiezopolska and Gabaldón, 2018). Fungal infections produce a spectrum of disorders, varying from life-threatening acute to less serious superficial infections (Warnock 2007). Unfortunately, because just a few kinds of antifungal agents, such as polyenes, azoles, etc. are available, the therapy choices for fungal diseases are highly restricted (Kathiravan et al., 2012). Antifungal drug tolerance procedures like efflux pump overexpression, biofilm generation, and other associated processes have evolved in these fungal pathogens, and they must be comprehended and explored (Scorzoni et al., 2017).

### 1.3 Need for natural products as potential antimicrobial agents

When antibiotics first became accessible 50 years ago, they were hailed as “wonder drugs,” but their widespread usage quickly led to abuse. Antimicrobial drugs have been losing their potency as a result of the development of drug tolerance during the previous few years (Saleem et al., 2010). Furthermore, because of the growth of the phenomenon of MDR in common human pathogenic organisms, the rapid appearance of novel diseases, and the possibility of using MDR agents in bioweapons, the requirement for novel antimicrobial drugs is higher than ever (Spellberg et al., 2004). As a result, there is an ongoing necessity to explore novel antimicrobials, especially since new medications are seldom released (Saleem et al., 2010). According to WHO report 2021, the pool of new antimicrobial compounds in clinical testing is limited. Only 6 of the thirty-two antibiotic agents in the clinical establishment that meet the WHO listing of critical pathogens were categorized as novel by WHO in the year 2019. Moreover, a lack of good-quality antimicrobial drugs remains a major concern. Antibiotic scarcities are impacting countries at all development stages, especially in the healthcare industry.

Tolerance to various antimicrobial agents among diverse pathogenic microbial species has become a key healthcare concern around. Owing to the drastic emergence of novel resistance processes and a decline in the efficacy of curing microbial infections, microbial responses to routine therapy fail, resulting in extended sickness, increased healthcare costs, and a high risk of mortality. Mostly all potential infecting organisms like pathogenic bacteria, fungi, etc. have used high degrees of MDR phenomenon, which has resulted in increased death and disease; therefore, they are known as “Super Bugs.” Since MDR is a natural process, improper antimicrobial drug usage, insufficient hygienic practices, improper food management, and inefficient infection prevention and control procedures all contribute to the establishment of MDR and increase its spread (Tanwar et al., 2014).

Multiple medical compounds of diverse chemical forms and bioactivity, such as antimicrobial, anticarcinogenic, and anti-inflammatory actions, were established as remedies and possess possible treatment implications for human diseases, thanks to natural products (Pham et al., 2019). Natural products are the secondary metabolites formed by a spectrum of species, including microorganisms, plants, animals, etc. Natural products act as both primary sources of novel chemical diversity and intrinsic components of currently available drug compilation. Many presently offered antifungal and antibacterial drugs, on the other hand, have unacceptable toxicity, and their extensive usage has resulted in the rapid development of drug-tolerant organisms, which are the major reason for the collapse in both agricultural and clinical settings (Saleem et al., 2010). Over a thousand microbial compounds were found so far, and most of those can be used for various medicinal purposes (Běhal 2001).

A large number of antimicrobial drugs derived from natural products with tremendous applications have been reported to enhance the value of natural products as a source of novel drug candidates against a wide range of human infectious diseases (Ye et al., 2020). Such different types of antimicrobial compounds obtained from various natural products were approved by the USFDA(US Food and Drug Administration) from 2000 to 2020 and the National Medical Products Administration (NMPA) of China, and include (in the clinical use): I) Natural antibacterial agents (Daptomycin, Fidaxomicin, Ertapenem sodium, etc); Natural antifungal agents (Caspofungin acetate, Micafungin sodium, Anidulafungin); Natural antiviral agents (Oseltamivir phosphate; Zanamivir; Enfuvirtide) (Ye et al., 2020).

#### 1.3.1 Plants and plant-derived products as a source of antimicrobial agents

**Fig. 5:**
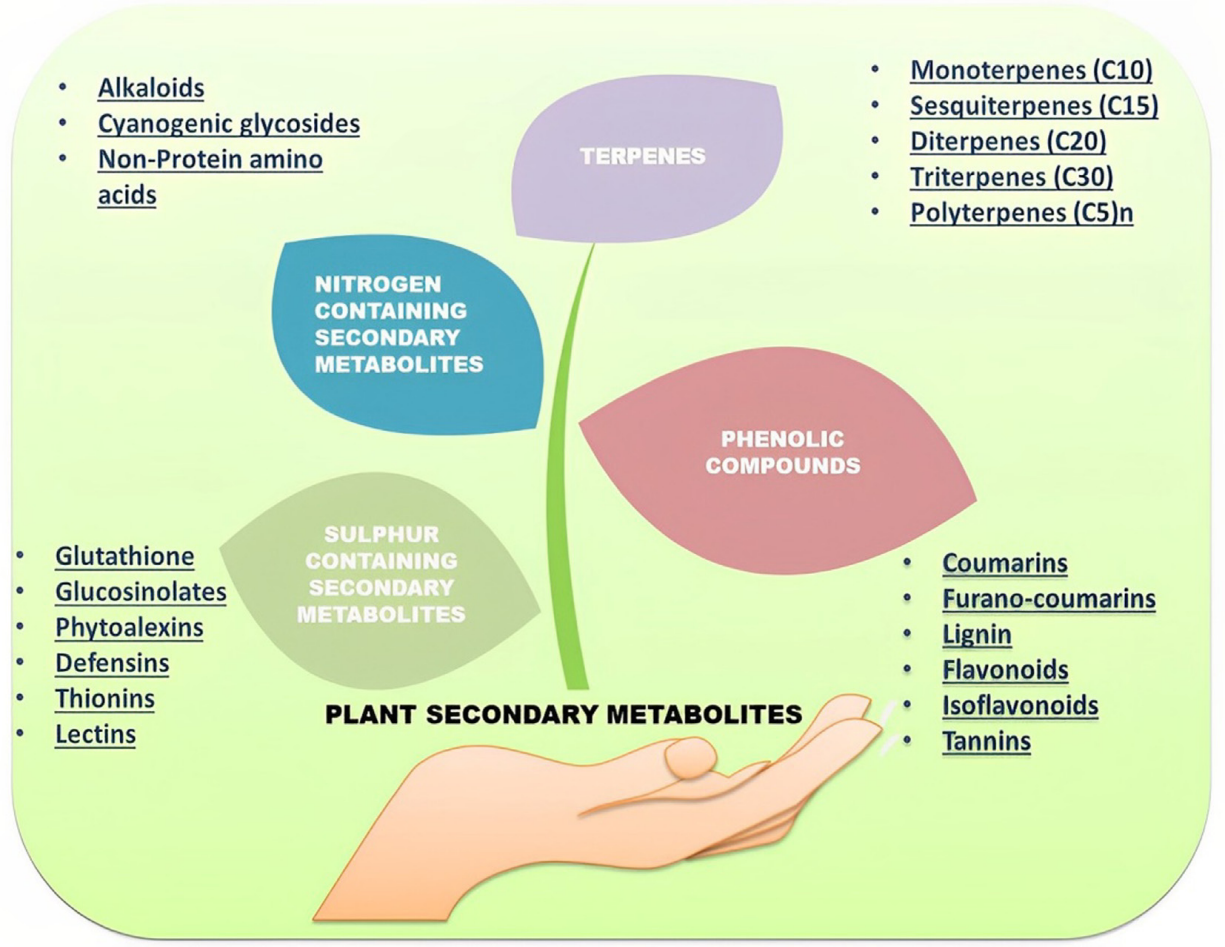
Diagrammatic representation of various natural products (Secondary Metabolites; Alkaloids, tannins, flavonoids, Sulphur-containing *sec*-metabolites, etc.) from plants as potential antimicrobial agents against different human pathogenic microbial organisms.

**Fig. 6:**
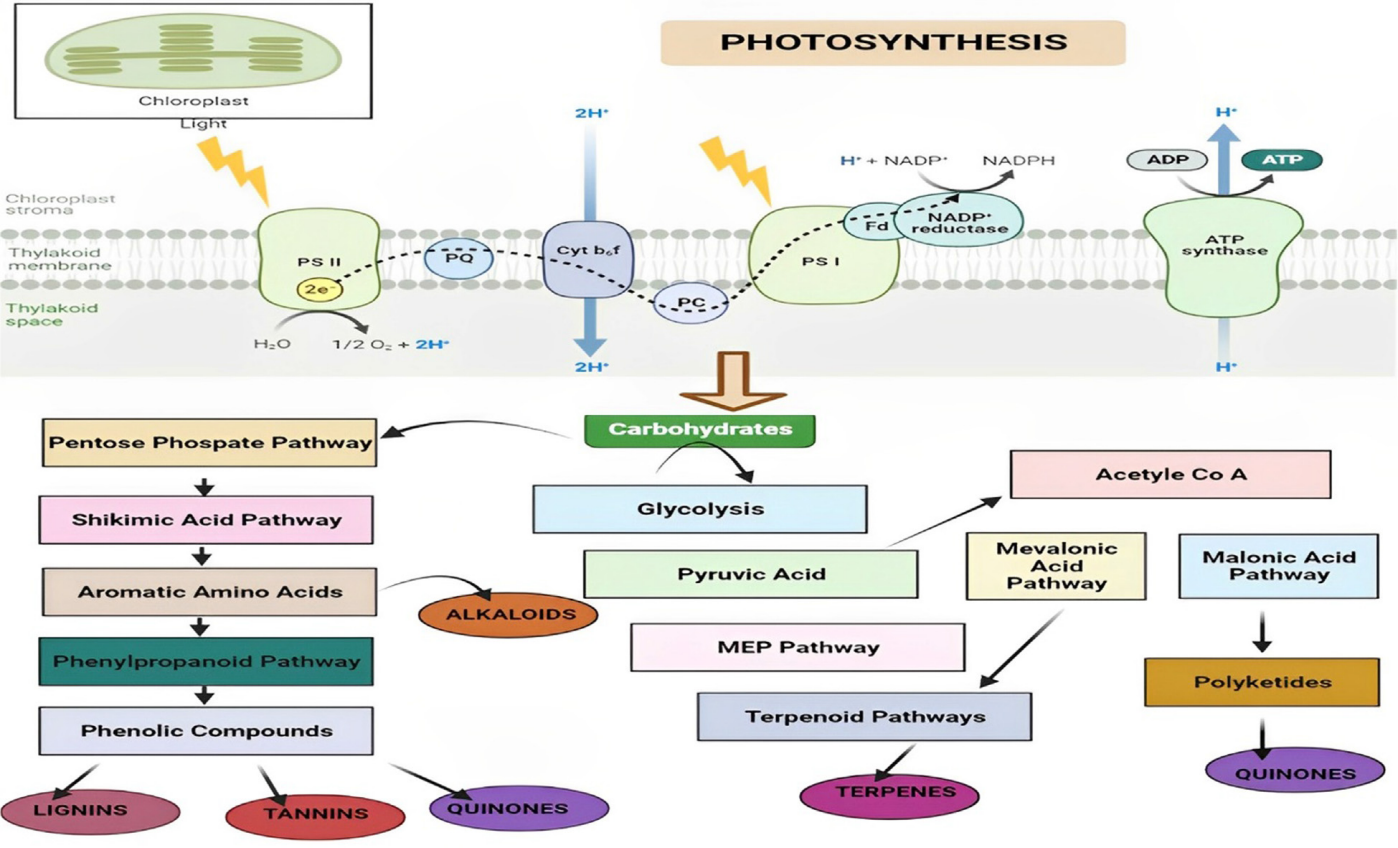
Diagrammatic illustration of biosynthetic pathways of different types of plant secondary metabolites (Alkaloids, Tannins, lignins, Quinones, etc). Plants adopt multiple pathways for the biosynthesis of various essential secondary metabolites. These useful secondary metabolites derived from plants perform an essential part in pharmaceutical and biomedical advancements.

Plants as medicine have been employed for a long time throughout the world. Medicinal plants include herbs, herbal components, and materials bearing several parts of plants or other plant-based compounds that are traditionally used to treat multiple health ailments (Petrovska 2012). Many countries across the world have evidence of medicinal plants being utilized to cure human sickness caused by numerous harmful microbes. For medicinal purposes, plants with recognized antimicrobial properties were employed. Some important plant products with antimicrobial properties have been listed in Table 1**.** They include a variety of biological substances that could be exploited to generate new medications to improve human health. Alkaloids, tannins, flavonoids, etc. **(**Fig. 5 **and** Fig. 6**)** are the phytochemical elements that act as defense processes against various microorganisms, like insects. Antibacterial, antifungal, anticancer, antioxidant, and other properties may be present in these substances (Jabborova et al., 2019).

**Table 1:** Some important plant products with numerous antimicrobial properties (Khameneh et al., 2019).

S.No	**Plant Product**	**Scientific Name**	**Antimicrobial Compound**	**Potency Against**
1.	Black pepper	*Piper nigrum*	Piperine	*Lactobacillus*, *Micrococcus,* different types of fungal species, etc.
2.	Cascara sagrada	*Rhamnus purshiana*	Tannins	Different types of fungal, Bacterial, and viral species.
3.	Onion	*Allium cepa*	Allicin	Different types of fungal and bacterial species.
4.	Thyme	*Thymus vulgaris*	Caffeic acid Thymol etc.	Different types of fungal, Bacterial, and viral species.
5.	Chamomile	*Matricaria chamomilla*	Anthemic acid	*M. tuberculosis*, *Staphylococcus. aureus* etc.
6.	Eucalyptus	*Eucalyptus globulus*	Tannin	Different types of Bacterial, and Viral species.
7.	Clove	*Syzygium aromaticum*	Eugenol	General
8.	Oregon grape	*Mahonia aquifolias*	Berberine	Plasmodium Trypansomes etc

Depending on their chemical structures, plant antimicrobial compounds are grouped into various categories including terpenoids, alkaloids, sulfur-containing compounds, and polyphenols (Khameneh et al., 2019). Some important natural compounds with tremendous antimicrobial characteristics are depicted in Table 2. The structural variety of substances generated from plants is enormous, and the antimicrobial property they exert against different pathogenic microbes is influenced by their structural arrangement. Phenolic molecules are amongst the most varied groups of secondary metabolites, with a spectrum of structural changes. The hydroxyl (–OH) groups of Phenolics are hypothesized to have an inhibitory impact (Lai and Roy 2004), since such compounds can communicate with bacterial membrane, disrupting membrane structures and causing cellular component leakages (Xue et al., 2013). Phenolic groups are complex, volatile, aromatic molecules located in a range of plant components, like glandular hairs, etc. in a wide range of chemical combinations (Fajinmi et al., 2019).

**Table 2:** Important plant natural compounds with potential antimicrobial properties (Khameneh et al., 2019).

S.No	**Natural Compound Category**	**Example**	**Potency against**	**Mechanism of Activity**
1.	Terpenes	i) Farnesol ii) Thymol iii) Menthol iv) Nerolidol	i) *S. aureus.* ii) *C. albicans, C. glabrata etc.* *iii) A. niger, A. fumigatus* etc. iv) *S. aureus*	i) Membrane disruption. ii) Disrupts membrane, inhibits efflux pump activity etc. iii) Membrane disruption. iv) Membrane disruption.
2.	Coumarins	i) Asphodelin A ii) 6-Geranyl coumarins iii) Galbanic acid	i) *S. aureus, C. albicans* etc. *ii) S. aureus.* *iii)* Multidrug resistant clinical isolates of *S. aureus*	i) Inhibitor of DNA gyrase. ii) Inhibits efflux pump activity. iii) Inhibits efflux pump activity.
3.	Alkaloids	i) Reserpine ii) Berberine iii) Piperine	i) *Staphylococcus* sp., *Streptococcus* sp.*,* etc. ii) *E. coli* etc*.* iii) *Staphylococcus aureus* etc*.*	i) Inhibits efflux pump activity. ii) Inhibits cell division, protein, and DNA synthesis. iii) Inhibits efflux pump activity.
4.	Phenolic compounds	i) Baicalein ii) Kaempferol iii) Rhamentin iv) Quercetin	i) *C. albicans* etc. *ii) C. albicans* etc. iii) *S. aureus* iv) *S. aureus*	i) Inhibits efflux pump activity. ii) Inhibits efflux pump activity. iii) Inhibits efflux pump activity. iv) Inhibits efflux pump activity.

Such chemical compounds are quite known for their antibacterial, antifungal, and other characteristics (Swamy et al., 2016). Flavonoids, quinones, tannins, lignans, and other naturally-derived polyphenolic chemicals have been documented to have antifungal activities (Lopes et al., 2017). For instance, many flavonols like kaempferol have demonstrated potent antifungal activities against various human pathogenic *Candida* species (Herrera et al., 2010). It has been found that Resveratrol, a natural phenolic compound possesses efflux pump inhibitor action in the case of various harmful bacteria (Klančnik et al., 2017, Khameneh et al., 2019). Baicalein is a flavone found in *Thymus vulgaris*, *Scutellaria baicalensis*, and *Scutellaria lateriflora* roots. *Scutellaria baicalensis* extract has been shown to have an antibacterial property in prior research studies (Lu et al., 2011). Piperine, a piperidine-based alkaloid, obtained from *Piper nigrum* and *Piper longum*, in association with ciprofloxacin, has been found to restrict the development of a mutant strain of *S. aureus* (Khan et al., 2006). Berberine is an isoquinoline alkaloid present in Berberis species' roots and stem bark. It's also the principal active component in *Rhizoma coptidis* and *Cortex phellodendri* and is being utilized in traditional medicine for centuries due to its antifungal, antibacterial, antiprotozoal, and antiviral properties (Khameneh et al., 2019). Coumarins are synthesized naturally by various plant species (Smyth et al., 2009). Multiple research studies have successfully displayed the antimicrobial action (antibacterial and antifungal) of both natural and synthetic by-products of coumarins (Melliou et al., 2005, Smyth et al., 2009). Moreover, terpenes/isoprenoids represent one of the most important diversified natural product families having both antibacterial and antifungal activities against various bacterial and fungal pathogens respectively (Khameneh et al., 2019). A range of terpenoid compounds has been discovered to have potent antimycotic activity in the case of *C. albicans* at safe doses (Smyth et al., 2009). Iridoids are found in various dicotyledonous plant families like Diervillaceae, Loganiacea, etc.

These plant-derived compounds possess many biological properties and also possess multiple antimicrobial characteristics (Tundis et al., 2008, Saleem et al., 2010, Di Gioia et al., 2020). A lignan, ((+)-lyoniresinol-3a-O-b-D-glucopyranoside), extracted from the root bark of *Lycium chinense*, has been found to possess potent antimicrobial properties against *Staphylococcus aureus* and many fungal pathogens e.g. *C. albicans,* etc (Saleem et al., 2010). In several *Candida* species, various plant essential oils were successfully tested to harbor effective antifungal characteristics. Sharifzadeh et al. discovered that essential oils from Trachyspermumammi have antifungal properties against several fluconazole-resistant *Candida* isolates (Sharifzadeh et al., 2015). In *C. albicans*, herbal essences from *Foeniculum vulgare*, *Saturejahortensis*, *Zataria multiflora,* etc. were tested for their antifungal action, and it was shown that the essential oils from *Z. multiflora* had significant antimicrobial properties (Gavanji et al., 2015).

**Fig. 7:**
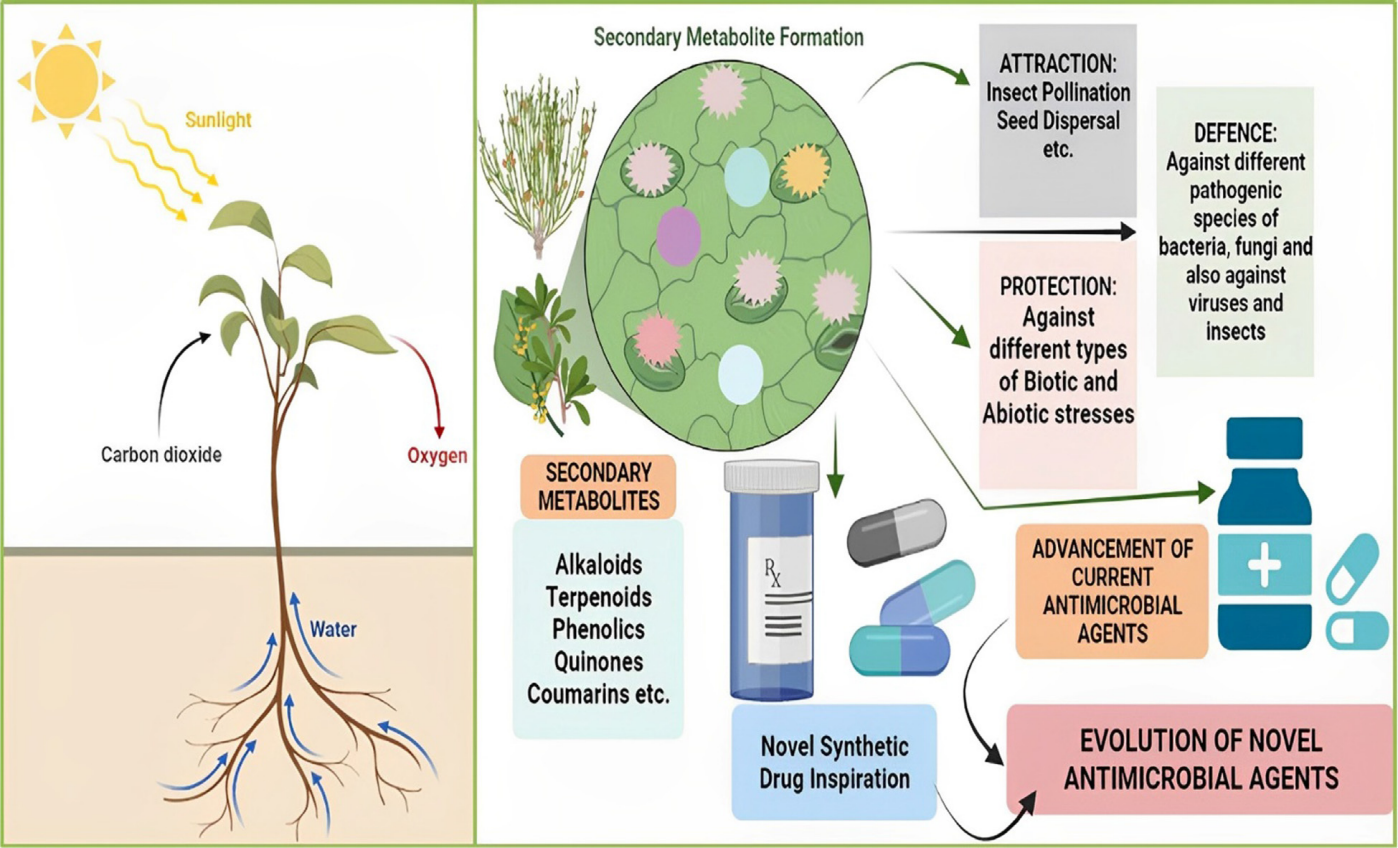
Schematic representation of the development of novel antimicrobial agents (Terpenoids, Alkaloids, Phenolics, Quinones, Coumarins, etc.) from different types of medicinal plants.

The findings imply that plants contain bioactive components in vast quantities. The process of identifying such compounds includes the separation, recognition, augmentation of pharmacokinetic and pharmacodynamic actions, and the selection of lead molecules for further therapeutic advancement **(**Fig. 7**)** (de Oliveira Santos et al., 2018). Furthermore, the combinative action of multiple plant extracts/bioactive molecules with conventional antimicrobial compounds or with other extracts/bioactive agents offers a promising therapeutic remedy against different clinical multidrug-resistant microbial organisms have been observed (Mukherjee et al., 2005).

##### 1.3.1.1 Mechanism of antimicrobial action of plant secondary metabolites

Secondary metabolites extracted from various plant species have multiple effects on microbial cells including impairment of membrane structure and dynamics function and structure, inhibition of nucleic acid (DNA/RNA) biosynthesis, interference with intermediary metabolism, stimulation of cytoplasmic constituent coagulation, and disruption of the cell communication process (Plaper et al., 2003, Parveen et al., 2004, Di Pasqua et al., 2006, Di Pasqua et al., 2007, Williams, 2007, Zhang et al., 2008, Bouhdid et al., 2010, Garvey et al., 2011, Radulovic et al., 2013). The interaction of the plant secondary metabolite with the cell membrane, followed by its diffusion via membrane (penetration into the cell interior), and finally its interaction with intracellular constituents/mechanisms are usually the events that occur in the case of antibacterial action (Sikkema et al., 1995). Plant-based natural products generally follow a similar pattern. A good example of a plant secondary metabolite is thymol, (monoterpene phenol) a potent plant secondary metabolite. This chemical is expected to be associated with both outer and inner cytoplasmic cell membranes by incorporated at the polar head group area of the lipid bilayer. This causes the cell membrane to flip, resulting in greater permeability and instability (Helander et al., 1998, Lambert et al., 2001, Walsh et al., 2003). Thymol, on the contrary, might perform a function in the up or downregulation of genes included in the outer membrane synthesis of proteins, inhibition of enzymes associated with thermal stress protection, ATP generation, and so on (Horváth et al., 2009, Di Pasqua et al., 2010). Trans-cinnamaldehyde, a shikimate metabolite, inhibits fungal cell-wall synthesizing enzymes by acting as a noncompetitive inhibitor of Beta-(1,3)-glucan synthase and a mixed inhibitor of chitin synthase isozymes (Bang et al., 2000). Trans-cinnamaldehyde also produced partial damage to the cytoplasmic membrane stability in *Saccharomyces cerevisiae*, resulting in excessive loss of essential cellular enzymes and molecules and finally leading to cell lysis (Di Pasqua et al., 2006).

The antimicrobial potency and method of the impact of various plant secondary metabolites could be affected and is extremely dependent on a variety of factors, including target cell characteristics, as well as the surroundings in which the antimicrobial activity must be demonstrated. The final effect of a plant's secondary metabolite or its mixture is heavily driven by environmental factors like hydrophilicity (water solubility) etc. (Denyer and Stewart 1998). The action of natural compounds on Gram-positive bacteria and fungal species is likely to be quite similar, with the key target being the cell membrane, whose destruction or alterations in permeability lead to the export of intracellular molecules and cytoplasm coagulation (Kalemba and Kunicka 2003). The cytoplasmic membrane is the principal target region of plant secondary metabolites, according to existing literature findings on antimicrobial action. Natural products display multiple effects on the dynamics, stability, and usefulness of the membrane. Some antifungal drugs, for example, communicate with ergosterol, the major fungal membrane sterol having a part in processes such as regulating membrane function and stability and regulating enzymes required for fungal cell development (Deva, 2002, Ahmad et al., 2011). Saponins (e.g., avenacins A-1, B-1, A-2, and B-2, a family of 4 structurally similar compounds with a common esterified trisaccharide component), several of which exhibit substantial antibacterial action (Osbourn 2003), might be used as an example. Saponins' antifungal activities are attributed to their capacity to bind with sterols in fungal membranes, resulting in pore development and impairment of membrane stability (Osbourn 2003). Carvacrol, an isomeric form of thymol, communicates with the cytoplasmic membrane by inserting between phospholipid acyl chains (Traditional, 1999, Ultee et al., 2000, Di Pasqua et al., 2006). As a result of the aforementioned process, the membrane fluidity is disrupted (increased), resulting in increased permeability. Increased permeability causes ion and ATP efflux, as well as a change in membrane potential and pH gradient (Ultee et al., 1999, Ultee et al., 2000). Eugenol, a phenylpropanoid present in many plant species, has a lytic impact on bacterial cells (Rhayour et al., 2003), and its mechanism of activity is non-specific membrane permeabilization (Hyldgaard et al., 2012), as evidenced by potassium and ATP efflux (Gill and Holley, 2006, Patwardhan et al., 2008). Eugenol is expected to interact with membrane proteins and inhibit or change their actions (Hyldgaard et al., 2012). A variety of additional plant metabolites are commonly found in antimicrobial plant extracts, such as linalool (Ait-Ouazzou et al., 2011), menthol (Trombetta et al., 2005), citral (Somolinos et al., 2010), etc. cause membrane permeabilization and subsequent processes. Limonene, on the contrary, alters cell shape and membrane permeability (Di Pasqua et al., 2006, Di Pasqua et al., 2007, Espina et al., 2011). Many reports have revealed that essential oils isolated from various plants impact the integrity of cell membranes (Radulovic et al., 2013). The essential oil of *Melaleuca alternifolia* haabours a remarkable antifungal property, which inspired Hammer et al. (Hammer et al., 2004) to examine its activity against *C. albicans*, *S. cerevisiae* etc. Tea tree oil alters the elaticity and consequently permeability of the fungal barrier, according to the study. Plant secondary metabolites' antibacterial activity can be aimed at intracellular processes including nucleic acid/protein synthesis and the process of cell communication in addition to cell membranes (Radulovic et al., 2013). This is the case with allicin, the active ingredient in garlic (*Allium sativum*) (Radulovic et al., 2013). Flavonoids disrupt both cytoplasmic membrane function and nucleic acid synthesis, making them one of the most active plant chemicals. Protein and RNA synthesis are also harmed, though to a smaller degree. Apigenin and quercetin, as well as a number of other flavonoids, have been discovered to inhibit DNA gyrase and other related properties (Cushnie and Lamb, 2005, Zhang et al., 2008).

##### 1.3.1.2 Economic and therapeutic potential of plant-derived antimicrobials

Natural products have seen a resurgence in popularity around the world. Consumers' assumption that natural products are superior; customer's unhappiness with conventional treatments; changes in laws enabling structure–function claims, leading to more permissive advertising; aging baby boomers; national concerns about healthcare expenditures have all contributed to this interest. From the standpoint of drug development as well as phytomedicines, the possibilities for turning antimicrobial compounds into therapeutics appear to be promising. Plant-based antimicrobial compounds provide direct commercial value to the herbal goods business (Ciocan and Bara 2007).

Microbial organisms are the source of most of the discovery and use of natural compounds as antimicrobial agents. Penicillin's discovery paved the way for antibiotics like aureomycin, streptomycin, and chloromycetin to follow (Trease and Evans 1972). Though soil bacteria or fungi synthesize the majority of therapeutically used antibiotics, higher plants also act as important antimicrobial sources (Trease and Evans 1972). Lichens' bacteriostatic and anti fungicidal capabilities, allinine's antibiotic action in Allium sativum (garlic), and berberines' antimicrobial action in goldenseal (Hydrastis canadensis) are some of the examples (Trease and Evans 1972).

Secondary metabolites like terpenoids, alkaloids, flavonoids, etc are present in abundance in plants. Antibacterial activities have been demonstrated for these substances derived from different types of herbs, spices, and plant extracts against various pathogenic microbes. As a consequence, there seems to be a surge in research on the antimicrobial characteristics of plant-derived substances, which could be utilized as a substitute for synthetic preservatives. Plant-derived antimicrobials are rather safe, and they could be utilized to extend the quality of foods to address food safety concerns (Bor et al., 2016).

#### 1.3.2 Mushrooms and algae as a source of antimicrobial agents

Mushrooms possess various antimicrobial and antioxidant properties among fungi (Gyawali and Ibrahim 2014). In vitro, extracts from wild *Laetiporus sulphureus* (Bull.) Murrill fruiting bodies have been found to show antimicrobial effects against various microbial pathogens like *C. albicans*, *C. parapsilopsis*, *S.aureus*, *Enterococcus faecalis,* etc. Edible mushroom extracts from Aphyllophorales (Ramesh and Pattar 2010), Agaricus (Öztürk et al., 2011), *Armillaria mellea*, *Paxillus involutus,* etc also have been found to show potent antimicrobial activity. The study done by Ramesh and Pattar (Ramesh and Pattar 2010) employed methanolic extracts from six wild edible mushrooms (*Ramaria formosa,* etc.). All of the isolates demonstrated high levels of phenols and flavonoids, having potent antimicrobial action against a variety of bacterial organisms (*S. aureus*, etc) and the human pathogenic fungi (*C. albicans*), indicating that the component concentrations have a direct impact on the isolated mushrooms' ability to fight various kinds of microbial pathogens. The fatty acids from *Agaricus essettei*, *A. bitorquis*, etc. were examined by ztürk et al. (Öztürk et al., 2011), who discovered that linoleic and palmitic acids were dominating and effective against different types of Gram-positive bacteria e.g *Bacillus subtilis*, *Bacillus cereus*, *Micrococcus flavus,* etc.

The antimicrobial characteristics of algae have been explored by many researchers (Quinto et al., 2019). (Herrero et al., 2013) explored such antimicrobial substances in macro-algae (*Himanthalia elongata*) and micro-algae (*Synechocystis* spp.) and found that the extracts from both possessed antimicrobial and antioxidant activities in the case of *E. coli* and *S. aureus* and. Another group of researchers (Devi et al., 2008) found similar results when they examined the extracts from *Haligra* spp. that were effective in the case of *S. aureus*. Antimicrobial activity was found in *Hymanthalia elongata*, *Laminaria digitate*, *Padina*, etc. against *Salmonella*, *Enterococcus faecalis, Pseudomonas aeruginosa,* etc. (Gupta et al., 2010, Dussault et al., 2016).

#### 1.3.3 Potential antimicrobials from Animal-derived products

Different types of antimicrobial compounds are known to be obtained from animals/animal products. Lactoferrin (Lf), (an iron-binding milk protein), harbors excellent antimicrobial characteristics against a spectrum of bacterial organisms and viruses (Lönnerdal 2011). In the USA, the protein has been recommended to be applied to beef and also as an antimicrobial agent in several meat-based products (Juneja et al., 2012). In many studies, Lf protein has been demonstrated to possess antimicrobial properties in the case of different foodborne microbes like *E.coli*, *Carnobacterium*, *Klebsiella*, *L. monocytogenes,* etc (Al-Nabulsi and Holley, 2005, Murdock et al., 2007). On the other hand, Chitosan, a polycationic biopolymer found naturally in the crustacean and arthropod exoskeletons represent one of the antimicrobial agents with numerous applications (Tikhonov et al., 2006). The antibacterial potential of various chitosans has been successfully investigated in the case of various Gram + ve and Gram –ve bacterial organisms (No et al., 2002). It has been found that water-soluble chitosan derivatives display tremendous antibacterial action in the case of *Staphylococcus. aureus*, *E. coli*, *Shigella dysenteriae*, *S. Typhimurium*, etc (Chung et al., 2011). Furthermore, the enzyme Lysozyme is found naturally in mammalian milk and avian eggs (Nazir et al., 2017). Lysozyme present in the eggs of hens represents a bacteriolytic enzyme well known for its broad-spectrum antimicrobial usage in the food industry (Tiwari et al., 2009). The enzyme has been fundamentally utilized to avoid late blowing defects in cheeses, caused by *Clostrodium tyrobutyricum*. Lysozyme displays excellent antimicrobial action in the case of *Listeria innocua* and *Saccharomyces cerevisiae* (Nazir et al., 2017). Certain milk-derived bioactive substances, like casein, are reported to harbor multifaceted capabilities, including antibacterial activities (Phelan et al., 2009). Antimicrobial potency of such peptides was demonstrated in the case of various pathogenic microbes, including Listeria, etc. (Fadaei 2012).

#### 1.3.4 Potential antimicrobial products derived from microbes

Alexander Fleming's discovery of penicillin (efficient against gram-positive bacteria) derived from *Penicillium notatum* in the year 1928 heralded a fundamental change in natural product sources from plants to microbes (Fleming, 2001, Tan and Tatsumura, 2015, Pham et al., 2019). Ever since the substances derived from microorganisms are used in medicinal, agricultural, and other related sectors (Sanchez et al., 2012). Polyketides are chemically varied natural compounds that are assembled by polyketide synthases (PKS). They are among the most essential metabolites for their therapeutic, agricultural, and industrial applications (Tae et al., 2007). Pikromycin, for instance, represents the first polyketide antibiotic discovered in *S. venezuelae* in the year 1950 (Jung et al., 2006). Pikromycin is particularly effective in the case of MDR respiratory pathogens (Woo et al., 2014). Erythromycin A, generated by *S. erythraea*, is another exceptional polyketide antibiotic with important clinical potential (Pham et al., 2019). The antibiotic is employed for treating various types of bacterial illnesses, including intestinal and respiratory infections., and is particularly useful in people who have had negative reactions to penicillin (Cobb et al., 2013). Antibiotics that bind to the rRNA subunit of the 30S bacterial ribosome and hinder the process of protein biosynthesis are categorized as aminoglycosides (Moazed and Noller, 1987, Pham et al., 2019). The aminoglycoside “Streptomycin” is the ist aminoglycoside discovered in the year 1944. It is produced by *S. griseus* and shows efficacy in the case of pulmonary tuberculosis (Pham et al., 2019). Aminoglycoside antibiotics like kanamycin, etc. have been found and broadly utilized since the discovery of streptomycin to treat pathogens that have evolved tolerance/resistance to streptomycin after continuous usage (Park et al., 2013).

In the year 1950, *Streptomyces noursei* produced nystatin, one of the first efficient polyene antifungal agents, showing activity against different *Aspergillus* species (Pham et al., 2019). In clinical practice, Nystatin is used as a topical antifungal to treat oral, gastrointestinal, and vaginal candidosis (Fjærvik and Zotchev 2005). Furthermore, Amphotericin B is a classical polyene antifungal agent derived from *Streptomyces nodosus* that is used to treat severe fungal diseases caused by various *Aspergillus* pathogenic species. It is particularly beneficial in individuals with organ transplants and other related conditions (Tevyashova et al., 2013). In a recent evaluation of natural compounds having antifungal properties against the human pathogenic fungi *C. albicans*, 71 of the 142 substances studied were shown to exhibit antifungal potency (Zida et al., 2017). The glycolipids (ieodoglucomide C and ieodoglycolipid) obtained from the marine bacterial organism *Bacillus licheniformis*, showed antifungal action against different pathogenic species like *C. albicans*, *Aspergillus niger*, *Colletotrichum acutatum*, etc. (Tareq et al., 2015, Pham et al., 2019).

#### 1.3.5 Peptides as promising antimicrobial agents

Antimicrobial peptides (AMPs), also referred to as host defense peptides, are small, usually positively charged peptides present in various life forms. Many AMPs can directly kill pathogenic microbes, whereas some operate indirectly by regulating various host defensive systems. Attempts to get AMPs into medical use are increasing against a backdrop of rapidly developing tolerance to standard antibiotic agents. AMPs are being studied in various medical settings as potential anti-infective agents and novel therapeutic drugs to modulate the immune system response, improve wound healing, etc. The rapid of AMPs possess excellent bactericidal action which renders them potential therapeutic anti-infective agents (Mahlapuu et al., 2016). A few AMPs have been approved for clinical usage to date, with polymyxins, which were first introduced in the 1950 s, being the best-known (Falagas et al., 2005, Zavascki et al., 2007, Landman et al., 2008). Polymyxins represent the last-resort medications for intravenous infection treatment associated with different drug-tolerant Gram-negative pathogenic species, but can also be used topically to prevent and treat local infections (Zavascki et al., 2007).

Presently, several AMPs are in the clinical development phase **(**Table 3**)** for cure and treatment in the case of different pathogenic bacterial organisms having omiganan and pexiganan, obtained from animal immune components, and synthetic LTX-109, majorly reported. Pexiganan, a twenty-two amino-acid membrane disruptor analog of magainin (Xenopus peptide), was assessed as a topical cream for the treatment of bacterial diseases in association with diabetic foot ulcers in 2 phase III clinical trials (Lipsky et al., 2008), besides other ongoing clinical trials (Mahlapuu et al., 2016).

**Table 3:** List of some important antimicrobial peptides and their respective clinical phases (Mahlapuu et al., 2016).

S.No	**Antimicrobial Peptides**	**Clinical Phase**	**Administration**	**Clinical Trial Identifier**
1.	Iseganan (IB-367)	Phase III	Oral solution	NCT00022373
2.	Pexiganan (MSI-78)	Phase III	Topical cream	NCT00563394,NCT00563433
3.	Omiganan	Phase II/III	Topical gel	NCT00231153,NCT01784133
4.	Lytixar (LTX-109)	Phase I/II	Topical Hydrogel	NCT01223222,NCT01803035,NCT01158235
5.	hLF1-11	Phase I/II	Intravenous treatment (in saline)	NCT00509938

Omiganan is a derivative of indolicidin, derived from bovine neutrophils, and was identified as a topical gel for the treatment of catheter-associated infections in several clinical trials. Moreover, LTX-109 is a synthetic antimicrobial peptidomimetic, assessed for local usage in uncomplicated Gram-positive skin-related diseases, S. aureus-related infections, etc. (Nilsson et al., 2015). Since a majority of the AMPs, together with the above-stated pexiganan, omiganan, and LTX-109, have been established for local usage/application, certain AMPs were also established for systemic administration. hLF1-11 represents a cationic part containing N-terminal amino acids one-eleven of human lactoferricin and has been established for the intravenous treatment of severe fungal and bacterial diseases in individuals with a weakened immune system (van der Velden et al., 2009). Besides hLF1-11, multiple additional AMPs have been evolved for the proper cure and treatment of various fungal diseases. For instance, novexatin, a cyclic and extremely cationic peptide based on human Alpha and Beta defensins is a potential antifungal target for serious toenail fungal infections (Fox 2013). Similarly, CZEN-002, a dimeric peptide obtained in sequence from Alpha-melanocyte-stimulating hormone, acts as a potent target in the case of vaginal candidiasis (Fjell et al., 2012).

Apart from administering AMPs directly, many efforts are underway to utilize agents to enhance the body's endogenous synthesis of AMPs to promote innate immune responses and thus treat diseases. Vitamin D3 has been found to directly affect the expression of many AMPs (Wang et al., 2004, Weber et al., 2005), and vitamin D supplements are now being investigated in multiple ongoing clinical trials for their usefulness to treat different types of bacterial infections (Yamshchikov et al., 2009).

**Fig. 8:**
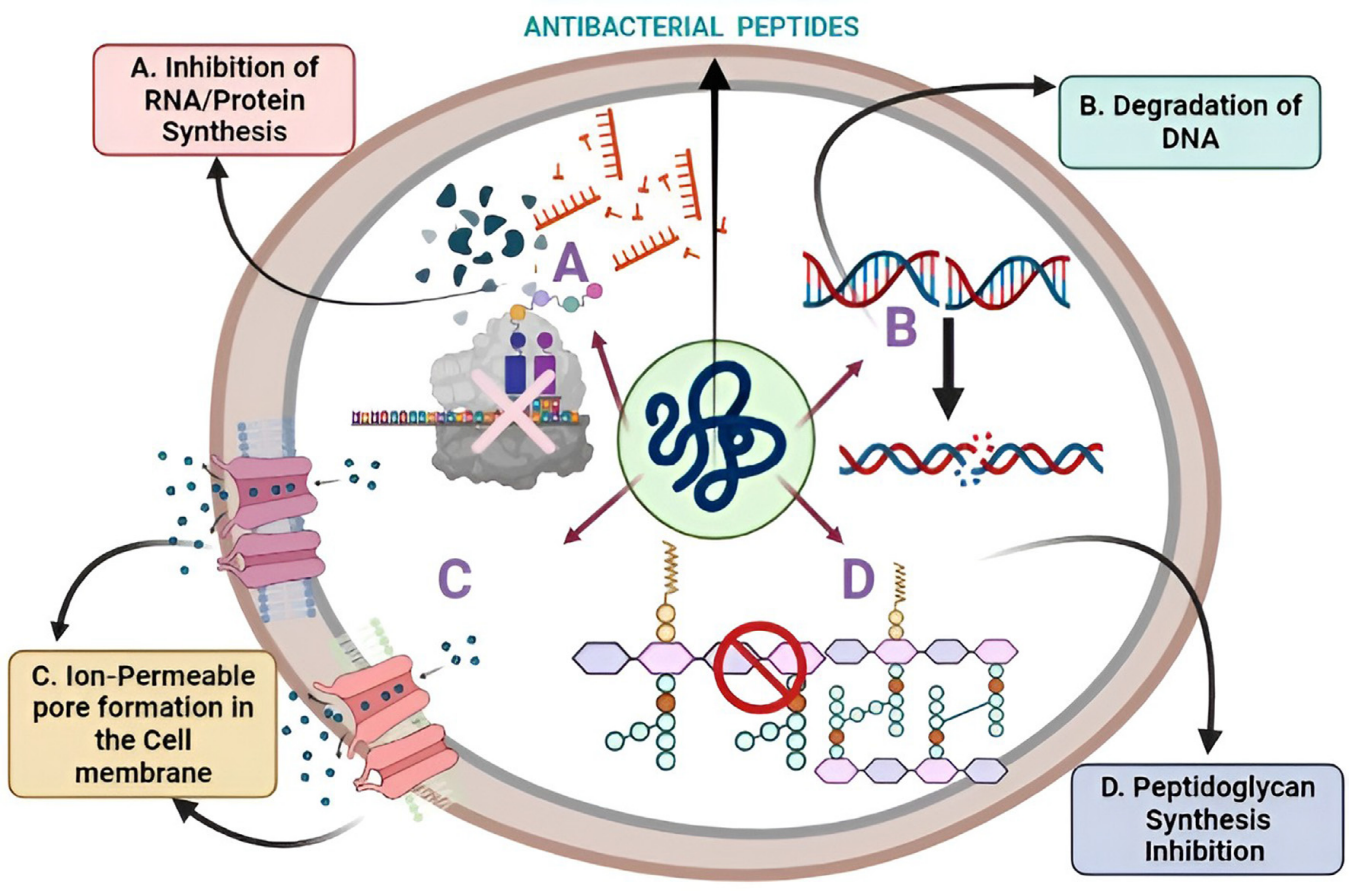
**I**mportant antimicrobial action mechanisms are depicted in this diagram. (A. Inhibition of RNA/Protein Synthesis; B. Degradation of DNA; C. Ion-Permeable pore formation in the Cell membrane; D. Peptidoglycan Synthesis suppression) of antibacterial peptides synthesized by various marine bacterial organisms.

A significant number of secondary metabolites of various forms, structures, and methods of antibacterial properties have been identified in a variety of marine bacterial organisms in recent times (Gokulan et al., 2014, Böhringer et al., 2017, Andryukov et al., 2018). Even though Gram-negative prokaryotes dominate the marine environment, manufacturers of antimicrobial peptides were mostly strains of Gram-positive marine bacteria, unlike those from terrestrial habitats (Mikhailov and Pivkin 2014, Giang et al., 2015, Andryukov et al., 2018, Wang and Lei, 2018). The majority of the identified antimicrobial metabolites possess the ability to destroy a broad spectrum of microorganisms in a short amount of time. Large antimicrobial proteins (>100 amino acids) are usually lytic proteins capable of binding nutrients or destroying certain cell patterns, triggering DNA breakdown and limiting intracellular peptidoglycan and protein production by affecting the organism's cell membrane structure/function (Pettit et al., 2010, Phelan et al., 2013, Rivetti et al., 2014, Giang et al., 2015, Chen et al., 2017, Gao et al., 2017, Andryukov et al., 2018, Kers et al., 2018, Andryukov et al., 2019) **(**Fig. 8**)**.

## 2 Conclusion and future perspectives

Microbial diseases have risen exponentially, with alarmingly high fatality rates. The proliferation of novel pathogens and the creation of new resistance patterns pose a threat to the elimination of infectious diseases. Antimicrobial drug efficacy has deteriorated to the point where it has become a global public health issue (Ye et al., 2020). Because of the rise in different drug-tolerant microbial organisms, drug toxicity, and other related factors, traditional antimicrobial agents are no longer effective. For the concerned individuals, new treatment options are urgently needed to control and eradicate the emerging microbial infections (Qadri et al., 2021, Qadri et al., 2022). The current COVID-19 crisis has also added to the drastic increase of such emerging microbial infections. Antimicrobial medications and remedies are still limited in their ability to address a range of pathogenic microbial diseases. These pathogenic bacterial and fungal species are resistant to presently offered antimicrobial agents and can adapt to a wide range of host environments, constituting a serious public health danger.

Natural products originating from natural sources like plants, microbes, etc. show great efficiency for treating infectious diseases, with fewer side effects, synergy, and the capacity to combat drug tolerance. Natural products are the most essential resources of modern therapeutics since they have a wide range of chemical and functional variability and are the bane of antibiotic tolerance (Ye et al., 2020). Natural products are a great source of physiologically active chemicals, and their role in drug discovery is well established. Natural products perform a vital role in drug development and continue to provide a significant number of new lead compounds. The two crucial elements that propel natural materials from precursors to medicines are pharmacological activity and druggability. Natural active chemicals are normally good lead compounds, although most of them struggle to meet the criteria. To address the present faults and limitations, such structural traits should be adjusted and improved (Franco and Vázquez, 2020, Ye et al., 2020).

There seems to be no significant recent part in the establishment of promising and innovative antimicrobial agents and strategies in this field. As a result, there is indeed a raising need for new remedial tactics and medications to combat such severe microbial infections. Improvements in the field of microbiology techniques in the coming decade should lead to the identification of novel antimicrobial agents which could benefit humanity. Furthermore, microbiology literacy is crucial for preventing the development of AMR. In conclusion, the review provides a complete overview of some vital natural products and their byproducts as antimicrobials against various human pathogenic bacterial and fungal species. Natural products serve as key sources of novel chemical diversity as well as integral components of currently accessible drug formulations. The review will be useful in refining current antimicrobial (antifungal and antibacterial) remedies as well as establishing new treatment strategies to tackle the rising number of bacterial and fungal diseases.

## Funding

The study was financially supported by grants to MAM by Science and Engineering Research Board, Department of Science and Technology (SERB-DST) Govt. of India, New Delhi; vide Project Grant No: TAR/001213/2018.

## Declaration of Competing Interest

The authors declare that they have no known competing financial interests or personal relationships that could have appeared to influence the work reported in this paper.

## References

[b0005] Ahmad A., Khan A., Akhtar F. (2011). Fungicidal activity of thymol and carvacrol by disrupting ergosterol biosynthesis and membrane integrity against Candida. Eur. J. Clin. Microbiol. Infect. Dis..

[b0010] Ait-Ouazzou A., Cherrat L., Espina L. (2011). The antimicrobial activity of hydrophobic essential oil constituents acting alone or in combined processes of food preservation. Innov. Food Sci. Emerg. Technol..

[b0015] Al-Nabulsi A.A., Holley R.A. (2005). Effect of bovine lactoferrin against Carnobacterium viridans. Food Microbiol..

[b0025] Andryukov B., Mikhaylov V., Besednova N. (2018). The bacteriocinogenic potential of marine microorganisms. Russ. J. Mar. Biol..

[b0030] Andryukov B., Mikhailov V., Besednova N. (2019). The biotechnological potential of secondary metabolites from marine bacteria. J. Marine Sci. Eng..

[b0035] Ayukekbong, J. A., M. Ntemgwa and A. N. Atabe, 2017. The threat of antimicrobial resistance in developing countries: causes and control strategies. Antimicrobial Resistance & Infection Control. 6, 1-8. 10.1186/s13756-017-0208-x PMC5433038 28515903

[b0040] Bang K.-H., Lee D.-W., Park H.-M. (2000). Inhibition of fungal cell wall synthesizing enzymes by trans-cinnamaldehyde. Biosci. Biotechnol. Biochem..

[b0045] Bassetti M., Peghin M., Timsit J.-F. (2016). The current treatment landscape: candidiasis. J. Antimicrob. Chemother..

[b0050] Běhal V. (2001). Nontraditional microbial bioactive metabolites. Folia Microbiol..

[b0055] Böhringer N., Fisch K.M., Schillo D. (2017). Antimicrobial potential of bacteria associated with marine sea slugs from North Sulawesi, Indonesia. Front. Microbiol..

[b0060] Bongomin F., Gago S., Oladele R.O. (2017). Global and multi-national prevalence of fungal diseases—estimate precision. J. Fungi.

[b0065] Bor, T., S. O. Aljaloud, R. Gyawali, et al., 2016. Antimicrobials from herbs, spices, and plants. Fruits, vegetables, and herbs, Elsevier: 551-578.

[b0070] Bouhdid S., Abrini J., Amensour M. (2010). Functional and ultrastructural changes in Pseudomonas aeruginosa and Staphylococcus aureus cells induced by Cinnamomum verum essential oil. J. Appl. Microbiol..

[b0075] Brown E.D., Wright G.D. (2016). Antibacterial drug discovery in the resistance era. Nature.

[b0080] Capozzi C., Volpi A., Maurici M. (2013). Healthcare-associated infections and antibiotic resistance: a global challenge for the 21st century. Igiene e Sanita Pubblica.

[b0085] Chen E., Chen Q., Chen S. (2017). Mathermycin, a lantibiotic from the marine actinomycete Marinactinospora thermotolerans SCSIO 00652. Appl. Environ. Microbiol..

[b0090] Chen N., Zhou M., Dong X. (2020). Epidemiological and clinical characteristics of 99 cases of 2019 novel coronavirus pneumonia in Wuhan, China: a descriptive study. The Lancet.

[b0095] Choi J.-S., Kim S., Motea E. (2017). Inhibiting translesion DNA synthesis as an approach to combat drug resistance to DNA damaging agents. Oncotarget.

[b0100] Chokshi A., Sifri Z., Cennimo D. (2019). Global contributors to antibiotic resistance. J. Global Infect. Dis..

[b0105] Chung Y.-C., Yeh J.-Y., Tsai C.-F. (2011). Antibacterial characteristics and activity of water-soluble chitosan derivatives prepared by the Maillard reaction. Molecules.

[b0110] Ciocan D., Bara I. (2007). Plant products as antimicrobial agents. Analele Stiintifice ale Universitatii “Alexandru Ioan Cuza” din Iasi Sec. II a. Genet. Biol. Mol..

[b0115] Cobb R.E., Luo Y., Freestone T. (2013).

[b0120] Cohen M.L. (2000). Changing patterns of infectious disease. Nature.

[b0125] Contou D., Claudinon A., Pajot O. (2020). Bacterial and viral co-infections in patients with severe SARS-CoV-2 pneumonia admitted to a French ICU. Ann. Intensive Care.

[b0130] Cupertino M.C., Resende M.B., Mayer N.A. (2020). Emerging and re-emerging human infectious diseases: a systematic review of the role of wild animals with a focus on public health impact. Asian Pacific J. Trop. Med..

[b0135] Cushnie T.T., Lamb A.J. (2005). Antimicrobial activity of flavonoids. Int. J. Antimicrob. Agents.

[b0140] da Silva J. (2016). DNA damage induced by occupational and environmental exposure to miscellaneous chemicals. Mutation Res./Rev. Mutation Res..

[b0145] de Oliveira Santos G.C., Vasconcelos C.C., Lopes A.J. (2018). Candida infections and therapeutic strategies: mechanisms of action for traditional and alternative agents. Front. Microbiol..

[b0150] Denyer S.P., Stewart G. (1998). Mechanisms of action of disinfectants. Int. Biodeterior. Biodegrad..

[b0155] Deva, R., 2002. Metabolism of arachidonic acid and formation of novel 3-hydroxyoxylipins of Candida albicans and interaction of Hela cells-Candida albicans as a model for vulvovaginal candidiasis: redundancy of signaling pathways for activation of COX-2.

[b0160] Devi K.P., Suganthy N., Kesika P. (2008). Bioprotective properties of seaweeds: in vitro evaluation of antioxidant activity and antimicrobial activity against food borne bacteria in relation to polyphenolic content. BMC Complement. Alternat. Med..

[b0165] Di Gioia S., Hossain M.N., Conese M. (2020). Biological properties and therapeutic effects of plant-derived nanovesicles. Open Med..

[b0170] Di Pasqua R., Hoskins N., Betts G. (2006). Changes in membrane fatty acids composition of microbial cells induced by addiction of thymol, carvacrol, limonene, cinnamaldehyde, and eugenol in the growing media. J. Agric. Food. Chem..

[b0175] Di Pasqua R., Betts G., Hoskins N. (2007). Membrane toxicity of antimicrobial compounds from essential oils. J. Agric. Food. Chem..

[b0180] Di Pasqua R., Mamone G., Ferranti P. (2010). Changes in the proteome of Salmonella enterica serovar Thompson as stress adaptation to sublethal concentrations of thymol. Proteomics.

[b0185] Doron S., Gorbach S. (2008). Bacterial infections: overview. Int. Encyclopedia Public Health.

[b0190] Dorr T., Vulic M., Lewis K. (2010). Ciprofloxacin causes persister formation by inducing the TisB toxin in. Escherichia coli.

[b0195] Dussault D., Vu K.D., Vansach T. (2016). Antimicrobial effects of marine algal extracts and cyanobacterial pure compounds against five foodborne pathogens. Food Chem..

[b0200] Espina L., Somolinos M., Lorán S. (2011). Chemical composition of commercial citrus fruit essential oils and evaluation of their antimicrobial activity acting alone or in combined processes. Food Control.

[b0205] Fadaei V. (2012). Milk Proteins-derived antibacterial peptides as novel functional food ingredients. Ann. Biol. Res..

[b0210] Fajinmi O., Kulkarni M., Benická S. (2019). Antifungal activity of the volatiles of Agathosma betulina and Coleonema album commercial essential oil and their effect on the morphology of fungal strains Trichophyton rubrum and T. mentagrophytes. S. Afr. J. Bot..

[b0215] Falagas M.E., Kasiakou S.K., Saravolatz L.D. (2005). Colistin: the revival of polymyxins for the management of multidrug-resistant gram-negative bacterial infections. Clin. Infect. Dis..

[b0220] Fjærvik, E. and S. B. Zotchev, 2005. Biosynthesis of the polyene macrolide antibiotic nystatin in Streptomyces noursei. Applied microbiology biotechnology. 67, 436-443. 10.1007/s00253-004-1802-4 15700127

[b0225] Fjell C.D., Hiss J.A., Hancock R.E. (2012). Designing antimicrobial peptides: form follows function. Nat. Rev. Drug Discovery.

[b0230] Fleming, A. J. B. o. t. W. H. O., 2001. On the antibacterial action of cultures of a penicillium, with special reference to their use in the isolation of B. influenzae. 79, 780-790. PMC2566493 11545337

[b0235] Fox J.L. (2013). Antimicrobial peptides stage a comeback: better understanding of the mechanisms of action, modification and synthesis of antimicrobial peptides is reigniting commercial development. Nat. Biotechnol..

[b0240] Franco C.M., Vázquez B.I. (2020). Natural compounds as antimicrobial agents. Multidiscip. Digital Publishing Inst..

[b0245] Gao X.-Y., Liu Y., Miao L.-L. (2017). Mechanism of anti-Vibrio activity of marine probiotic strain Bacillus pumilus H2, and characterization of the active substance. AMB Express.

[b0250] Garvey M.I., Rahman M.M., Gibbons S. (2011). Medicinal plant extracts with efflux inhibitory activity against Gram-negative bacteria. Int. J. Antimicrob. Agents.

[b0255] Gavanji S., Zaker S.R., Nejad Z.G. (2015). Comparative efficacy of herbal essences with amphotricin B and ketoconazole on Candida albicans in the in vitro condition. Integr. Med. Res..

[b0260] Nguyen Dang Giang, C., Z. Sebesvari, F. Renaud, et al., 2015. Occurrence and dissipation of the antibiotics sulfamethoxazole, sulfadiazine, trimethoprim, and enrofloxacin in the Mekong Delta, Vietnam. Plos one. 10, e0131855. 10.1371/journal.pone.0131855 PMC4489625 26135396

[b0265] Gill A., Holley R. (2006). Disruption of Escherichia coli, Listeria monocytogenes and Lactobacillus sakei cellular membranes by plant oil aromatics. Int. J. Food Microbiol..

[b0270] Gokulan, K., S. Khare and C. Cerniglia, 2014. METABOLIC PATHWAYS| production of secondary metabolites of bacteria.

[b0275] Gu S., Chen Y., Wu Z. (2020). Alterations of the gut microbiota in patients with coronavirus disease 2019 or H1N1 influenza. Clin. Infect. Dis..

[b0280] Gupta A., Gupta R., Singh R.L. (2017).

[b0285] Gupta S., Rajauria G., Abu-Ghannam N. (2010). Study of the microbial diversity and antimicrobial properties of Irish edible brown seaweeds. Int. J. Food Sci. Technol..

[b0290] Gyawali R., Ibrahim S.A. (2014). Natural products as antimicrobial agents. Food Control.

[b0295] Hammer K., Carson C., Riley T. (2004). Antifungal effects of Melaleuca alternifolia (tea tree) oil and its components on Candida albicans, Candida glabrata and Saccharomyces cerevisiae. J. Antimicrob. Chemother..

[b0300] Haque M., McKimm J., Sartelli M. (2020). Strategies to prevent healthcare-associated infections: a narrative overview. Risk Manage. Healthcare Policy.

[b0305] Helander I.M., Alakomi H.-L., Latva-Kala K. (1998). Characterization of the action of selected essential oil components on Gram-negative bacteria. J. Agric. Food. Chem..

[b0310] Herrera C.L., Alvear M., Barrientos L. (2010). The antifungal effect of six commercial extracts of Chilean propolis on Candida spp. Ciencia Investig. Agraria.

[b0315] Herrero M., Mendiola J.A., Plaza M. (2013).

[b0320] Horváth G., Kovács K., Kocsis B. (2009). Effect of thyme (Thymus vulgaris L.) essential oil and its main constituents on the outer membrane protein composition of Erwinia strains studied with microfluid chip technology. Chromatographia.

[b0325] Hyldgaard M., Mygind T., Meyer R.L. (2012). Essential oils in food preservation: mode of action, synergies, and interactions with food matrix components. Front. Microbiol..

[b0330] Jabborova D., Davranov K., Egamberdieva D. (2019).

[b0335] Juneja, V. K., H. P. Dwivedi and X. Yan, 2012. Novel natural food antimicrobials. Annual review of food science technology. 3, 381-403. 10.1146/annurev-food-022811-101241 22385168

[b0340] Jung, W. S., S. K. Lee, J. S. J. Hong, et al., 2006. Heterologous expression of tylosin polyketide synthase and production of a hybrid bioactive macrolide in Streptomyces venezuelae. 72, 763-769. 10.1007/s00253-006-0318-5 16493552

[b0345] Kalemba D., Kunicka A. (2003). Antibacterial and antifungal properties of essential oils. Curr. Med. Chem..

[b0350] Kapoor G., Saigal S., Elongavan A. (2017). Action and resistance mechanisms of antibiotics: a guide for clinicians. J. Anaesthesiol., Clin. Pharma..

[b0355] Kathiravan, M. K., A. B. Salake, A. S. Chothe, et al., 2012. The biology and chemistry of antifungal agents: a review. Bioorganic medicinal chemistry. 20, 5678-5698.. 10.1016/j.bmc.2012.04.045 22902032

[b0360] Kers J.A., Sharp R.E., Defusco A.W. (2018). Mutacin 1140 lantibiotic variants are efficacious against Clostridium difficile infection. Front. Microbiol..

[b0365] Khameneh, B., M. Iranshahy, V. Soheili, et al., 2019. Review on plant antimicrobials: A mechanistic viewpoint. Antimicrobial Resistance Infection Control. 8, 1-28. 10.1186/s13756-019-0559-6 PMC6636059 31346459

[b0370] Khan, I. A., Z. M. Mirza, A. Kumar, et al., 2006. Piperine, a phytochemical potentiator of ciprofloxacin against Staphylococcus aureus. Antimicrobial agents chemotherapy. 50, 810-812. 10.1128/AAC.50.2.810-812.2006 PMC1366922 16436753

[b0375] Klančnik A., Šikić Pogačar M., Trošt K. (2017). Anti-Campylobacter activity of resveratrol and an extract from waste Pinot noir grape skins and seeds, and resistance of Camp. jejuni planktonic and biofilm cells, mediated via the Cme ABC efflux pump. J. Appl. Microbiol..

[b0380] Ksiezopolska E., Gabaldón T. (2018). Evolutionary emergence of drug resistance in Candida opportunistic pathogens. Genes.

[b0385] Lai C.-C., Chen S.-Y., Ko W.-C. (2021). Increased antimicrobial resistance during the COVID-19 pandemic. Int. J. Antimicrob. Agents.

[b0390] Lai P., Roy J. (2004). Antimicrobial and chemopreventive properties of herbs and spices. Curr. Med. Chem..

[b0395] Lambert R., Skandamis P.N., Coote P.J. (2001). A study of the minimum inhibitory concentration and mode of action of oregano essential oil, thymol and carvacrol. J. Appl. Microbiol..

[b0400] Landman D., Georgescu C., Martin D.A. (2008). Polymyxins revisited. Clin. Microbiol. Rev..

[b0405] Lewis K. (2007). Persister cells, dormancy and infectious disease. Nat. Rev. Microbiol..

[b0410] Li J., Wang J., Yang Y. (2020). Etiology and antimicrobial resistance of secondary bacterial infections in patients hospitalized with COVID-19 in Wuhan, China: a retrospective analysis. Antimicrob. Resist. Infect. Control..

[b0415] Lipsky B.A., Holroyd K.J., Zasloff M. (2008). Topical versus systemic antimicrobial therapy for treating mildly infected diabetic foot ulcers: a randomized, controlled, double-blinded, multicenter trial of pexiganan cream. Clin. Infect. Dis..

[b0420] Lobie T.A., Roba A.A., Booth J.A. (2021). Antimicrobial resistance: A challenge awaiting the post-COVID-19 era. Int. J. Infect. Dis..

[b0425] Lönnerdal, B., 2011. Biological effects of novel bovine milk fractions. Milk milk products in human nutrition. 67, 41-54. 10.1159/000325574 21335989

[b0430] Lopes G., Pinto E., Salgueiro L. (2017). Natural products: an alternative to conventional therapy for dermatophytosis?. Mycopathologia.

[b0435] Lu, Y., R. Joerger and C. Wu, 2011. Study of the chemical composition and antimicrobial activities of ethanolic extracts from roots of Scutellaria baicalensis Georgi. Journal of agricultural food chemistry. 59, 10934-10942. 10.1021/jf202741x 21866919

[b0440] Mahlapuu, M., J. Håkansson, L. Ringstad, et al., 2016. Antimicrobial peptides: an emerging category of therapeutic agents. Frontiers in cellular infection microbiology. 6, 194. 10.3389/fcimb.2016.00194 PMC5186781 28083516

[b0445] Manesh A., Varghese G.M. (2021). Rising antimicrobial resistance: an evolving epidemic in a pandemic. Lancet Microbe..

[b0495] Melliou E., Magiatis P., Mitaku S. (2005). Natural and synthetic 2, 2-dimethylpyranocoumarins with antibacterial activity. J. Nat. Prod..

[b0500] Mikhailov V., Pivkin M. (2014). The study of marine bacteria and fungi. Some results and trends for researching. Vestn. Dal’nevost. Otd. Ross. Akad. Nauk..

[b0505] Mir M. (2015). Introduction to costimulation and costimulatory molecules. Develop. Costimulatory Mol. Immunother. Dis..

[b0510] Mir M.A. (2022).

[b0515] Mir M.A., Agrewala J.N. (2007). Influence of CD80 and CD86 co-stimulation in the modulation of the activation of antigen presenting cells. Curr. Immunol. Rev..

[b0530] Mir Ahmed Manzoor *, Mehraj Umar (2019). Double-crosser of the Immune System: Macrophages in Tumor Progression and Metastasis. Current Immunology Reviews.

[b0475] Mir M.A., Aisha S., Qadri H. (2022). Chapter 2 - Evolution of antimicrobial drug resistance in human pathogenic bacteria. Human Pathogenic Microbes.

[b0520] Mir M.A., Al-baradie R. (2013). Tuberculosis time bomb-a global emergency: need for alternative vaccines. J. Health Sci..

[b0525] Mir M.A., Albaradie R.S. (2014). Inflammatory mechanisms as potential therapeutic targets in stroke. Adv. Neuroimmune Biol..

[b0450] Mir M.A., Hamdani S.S., Qadri H. (2022). Chapter 6 - Significance of immunotherapy for human bacterial diseases and antibacterial drug discovery. Human Pathogenic Microbes.

[b0485] Mir M.A., Jan U., Qadri H. (2022). Chapter 7 - Significance of immunotherapy for human fungal diseases and antifungal drug discovery. Human Pathogenic Microbes.

[b0465] Mir M.A., Kumawat M., Nabi B. (2022). Chapter 8 - Combinatorial approach to combat drug resistance in human pathogenic bacteria. Human Pathogenic Microbes.

[b0480] Mir M.A., Qadri H., Aisha S. (2022). Chapter 5 - Combating human fungal infections: need for new antifungal drugs and therapies. Human Pathogenic Microbes.

[b0490] Mir M.A., Rasool U., Aisha S. (2022). Chapter 1 - Human pathogenic microbes (bacterial and fungal) and associated diseases. Human Pathogenic Microbes.

[b0455] Mir M.A., Usman M., Qadri H. (2022). Chapter 10 - Recent trends in the development of bacterial and fungal vaccines. Human Pathogenic Microbes.

[b0540] Moazed D., Noller H.F.J.N. (1987). Interaction of antibiotics with functional sites in 16S ribosomal. RNA.

[b0545] Mohamed A., Hassan T., Trzos-Grzybowska M. (2021). Multi-triazole-resistant Aspergillus fumigatus and SARS-CoV-2 co-infection: a lethal combination. Med. Mycol. Case Rep..

[b0550] Moloney M.G. (2016). Natural products as a source for novel antibiotics. Trends Pharmacol. Sci..

[b0555] Mukherjee P.K., Sheehan D.J., Hitchcock C.A. (2005). Combination treatment of invasive fungal infections. Clin. Microbiol. Rev..

[b0560] Murdock C., Cleveland J., Matthews K. (2007). The synergistic effect of nisin and lactoferrin on the inhibition of Listeria monocytogenes and Escherichia coli O157: H7. Lett. Appl. Microbiol..

[b0565] Nazir F., Salim R., Yousf N. (2017). Natural antimicrobials for food preservation. Pharmacogn. Phytochem..

[b0570] Netea M.G., Giamarellos-Bourboulis E.J., Domínguez-Andrés J. (2020). Trained immunity: a tool for reducing susceptibility to and the severity of SARS-CoV-2 infection. Cell.

[b0575] Nicholson L.B. (2016). The immune system. Essays Biochem..

[b0535] Mir, Manzoor A., et al. "Nanomedicine in Human Health Therapeutics and Drug Delivery: Nanobiotechnology and Nanobiomedicine." *Applications of Nanomaterials in Agriculture, Food Science, and Medicine,* edited by Mohd Amin Bhat, et al., IGI Global, 2021, pp. 229-251. 10.4018/978-1-7998-5563-7.ch013

[b0580] Nilsson, A. C., H. Janson, H. Wold, et al., 2015. LTX-109 is a novel agent for nasal decolonization of methicillin-resistant and-sensitive Staphylococcus aureus. Antimicrobial agents chemotherapy. 59, 145-151. 10.1128/AAC.03513-14 PMC4291342 25331699

[b0585] No H.K., Park N.Y., Lee S.H. (2002). Antibacterial activity of chitosans and chitosan oligomers with different molecular weights. Int. J. Food Microbiol..

[b0590] O'Neill, J., 2016. Tackling drug-resistant infections globally: final report and recommendations.

[b0595] Organization, W. H., 2001. WHO global strategy for containment of antimicrobial resistance, World Health Organization.

[b0600] Organization, W. H., 2012. The evolving threat of antimicrobial resistance: options for action, World Health Organization.

[b0605] Organization, W. H., 2014. Antimicrobial resistance: global report on surveillance, World Health Organization.

[b0610] Osbourn A.E. (2003). Saponins in cereals. Phytochemistry.

[b0615] Öztürk M., Duru M.E., Kivrak Ş. (2011). In vitro antioxidant, anticholinesterase and antimicrobial activity studies on three Agaricus species with fatty acid compositions and iron contents: a comparative study on the three most edible mushrooms. Food Chem. Toxicol..

[b0620] Park S.R., Park J.W., Ban Y.H. (2013). 2-Deoxystreptamine-containing aminoglycoside antibiotics: recent advances in the characterization and manipulation of their biosynthetic pathways. Nat. Prod. Rep..

[b0625] Parveen M., Hasan M.K., Takahashi J. (2004). Response of Saccharomyces cerevisiae to a monoterpene: evaluation of antifungal potential by DNA microarray analysis. J. Antimicrob. Chemother..

[b0630] Patwardhan B., Vaidya A.D., Chorghade M. (2008). Reverse pharmacology and systems approaches for drug discovery and development. Curr. Bioact. Compd..

[b0635] Payne D.J., Miller L.F., Findlay D. (2015). Time for a change: addressing R&D and commercialization challenges for antibacterials. Philos. Trans. Royal Soc. B: Biol. Sci..

[b0640] Petrovska B.B. (2012). Historical review of medicinal plants’ usage. Pharmacogn. Rev..

[b0645] Pettit G.R., Ye Q., Herald D.L. (2010). Antineoplastic agents. 573. Isolation and structure of papilistatin from the papilionid butterfly Byasa polyeuctes termessa. J. Nat. Prod..

[b0650] Pham, J. V., M. A. Yilma, A. Feliz, et al., 2019. A review of the microbial production of bioactive natural products and biologics. 10, 1404. 10.3389/fmicb.2019.01404 PMC6596283 31281299

[b0655] Phelan M., Aherne A., FitzGerald R.J. (2009). Casein-derived bioactive peptides: biological effects, industrial uses, safety aspects and regulatory status. Int. Dairy J..

[b0660] Phelan R.W., Barret M., Cotter P.D. (2013). Subtilomycin: a new lantibiotic from Bacillus subtilis strain MMA7 isolated from the marine sponge Haliclona simulans. Mar. Drugs.

[b0665] Pidot S.J., Gao W., Buultjens A.H. (2018). Increasing tolerance of hospital Enterococcus faecium to handwash alcohols. Sci. Transl. Med..

[b0670] Plaper A., Golob M., Hafner I. (2003). Characterization of quercetin binding site on DNA gyrase. Biochem. Biophys. Res. Commun..

[b0675] Posteraro B., Torelli R., Vella A. (2020). Pan-echinocandin-resistant Candida glabrata bloodstream infection complicating COVID-19: a fatal case report. J. Fungi.

[b0680] Prestinaci, F., P. Pezzotti and A. Pantosti, 2015. Antimicrobial resistance: a global multifaceted phenomenon. Pathogens and global health 109, 309-318. 10.1179/2047773215Y.0000000030 PMC4768623 26343252

[b0685] Prestinaci F., Pezzotti P., Pantosti A. (2015). Antimicrobial resistance: a global multifaceted phenomenon. Pathog. Global Health.

[b0690] Qadri H., Haseeb A., Mir M. (2021). Novel strategies to combat the emerging drug resistance in human pathogenic microbes. Curr. Drug Targets.

[b0695] Qadri H., Qureshi M.F., Mir M.A. (2021). Glucose-The X Factor for the survival of human fungal pathogens and disease progression in the host. Microbiol. Res..

[b0700] Qadri H., Shah A.H., Mir M.A. (2022).

[b0705] Quinto E.J., Caro I., Villalobos-Delgado L.H. (2019). Food safety through natural antimicrobials. Antibiotics.

[b0710] Radulovic N., Blagojevic P., Stojanovic-Radic Z. (2013). Antimicrobial plant metabolites: structural diversity and mechanism of action. Curr. Med. Chem..

[b0715] Ramesh C., Pattar M.G. (2010). Antimicrobial properties, antioxidant activity and bioactive compounds from six wild edible mushrooms of western ghats of Karnataka. India. Pharmacogn. Res..

[b0720] K. Redhu, A., A. H. Shah and R. Prasad, 2016. MFS transporters of Candida species and their role in clinical drug resistance. FEMS yeast research. 16, fow043. 10.1093/femsyr/fow043 27188885

[b0725] Reygaert W.C. (2018). An overview of the antimicrobial resistance mechanisms of bacteria. AIMS Microbiol..

[b0730] Rhayour K., Bouchikhi T., Tantaoui-Elaraki A. (2003). The mechanism of bactericidal action of oregano and clove essential oils and of their phenolic major components on Escherichia coli and Bacillus subtilis. J. Essent. Oil Res..

[b0735] Rivetti I., Fraschetti S., Lionello P. (2014). Global warming and mass mortalities of benthic invertebrates in the Mediterranean Sea. PLoS ONE.

[b0740] Rossiter S.E., Fletcher M.H., Wuest W.M. (2017). Natural products as platforms to overcome antibiotic resistance. Chem. Rev..

[b0745] Saleem M., Nazir M., Ali M.S. (2010). Antimicrobial natural products: an update on future antibiotic drug candidates. Nat. Prod. Rep..

[b0750] Sanchez, S., S. Guzman-Trampe, M. Ávalos, et al., 2012. Microbial natural products, John Wiley & Sons, Inc.: Hoboken, NJ.

[b0755] Scorzoni, L., A. C. de Paula e Silva, C. M. Marcos, et al., 2017. Antifungal therapy: new advances in the understanding and treatment of mycosis. Frontiers in microbiology. 8, 36. 10.3389/fmicb.2017.00036 PMC5253656 28167935

[b0760] Sharifzadeh A., Khosravi A., Shokri H. (2015). Antifungal effect of Trachyspermum ammi against susceptible and fluconazole-resistant strains of Candida albicans. J. Mycol. Med..

[b0765] Sheikh B.A., Bhat B.A., Alshehri B. (2021). Nano-drug delivery systems: possible end to the rising threats of tuberculosis. J. Biomed. Nanotechnol..

[b0770] Sheikh, B. A., B. A. Bhat, Z. Ahmad, et al., 2021. Strategies employed to evade the host immune response and the mechanism of drug resistance in Mycobacterium tuberculosis: In search of finding new targets. Current pharmaceutical biotechnology. 10.2174/1389201023666211222164938 34951359

[b0775] Sikkema J., de Bont J.A., Poolman B. (1995). Mechanisms of membrane toxicity of hydrocarbons. Microbiol. Rev..

[b0780] Smyth T., Ramachandran V., Smyth W. (2009). A study of the antimicrobial activity of selected naturally occurring and synthetic coumarins. Int. J. Antimicrob. Agents.

[b0785] Somolinos M., García D., Condón S. (2010). Inactivation of Escherichia coli by citral. J. Appl. Microbiol..

[b0790] Spellberg B., Powers J.H., Brass E.P. (2004). Trends in antimicrobial drug development: implications for the future. Clin. Infect. Dis..

[b0795] Swamy, M. K., M. S. Akhtar and U. R. Sinniah, 2016. Antimicrobial properties of plant essential oils against human pathogens and their mode of action: an updated review. Evidence-Based Complementary. 10.1155/2016/3012462 PMC5206475 28090211

[b0800] Tae, H., E.-B. Kong and K. J. B. b. Park, 2007. ASMPKS: an analysis system for modular polyketide synthases. 8, 1-9. 10.1186/1471-2105-8-327 PMC2008767 17764579

[b0805] Tagkopoulos I. (2019). Benzalkonium chlorides: uses, regulatory status, and microbial resistance. Appl. Environ. Microbiol..

[b0810] Tan, S. Y. and Y. J. S. m. j. Tatsumura, 2015. Alexander Fleming (1881–1955): discoverer of penicillin. 56, 366. 10.11622/smedj.2015105 PMC4520913 26243971

[b0815] Tanwar, J., S. Das, Z. Fatima, et al., 2014. Multidrug resistance: an emerging crisis. Interdisciplinary perspectives on infectious diseases. 2014, 10.1155/2014/541340 PMC4124702 25140175

[b0820] Tareq F.S., Lee H.S., Lee Y.J. (2015). Ieodoglucomide C and Ieodoglycolipid, New Glycolipids from a Marine-Derived Bacterium Bacillus licheniformis 09IDYM23. Lipids.

[b0825] Tevyashova, A. N., E. N. Olsufyeva, S. E. Solovieva, et al., 2013. Structure-antifungal activity relationships of polyene antibiotics of the amphotericin B group. Antimicrobial agents chemotherapy. 57, 3815-3822. 10.1128/AAC.00270-13 PMC3719701 23716057

[b0830] Tikhonov V.E., Stepnova E.A., Babak V.G. (2006). Bactericidal and antifungal activities of a low molecular weight chitosan and its N-/2 (3)-(dodec-2-enyl) succinoyl/-derivatives. Carbohydr. Polym..

[b0835] Tiwari, B. K., V. P. Valdramidis, C. P. O’Donnell, et al., 2009. Application of natural antimicrobials for food preservation. Journal of agricultural food chemistry. 57, 5987-6000. 10.1021/jf900668n 19548681

[b0840] Traditional W. (1999).

[b0845] Trease G., Evans W. (1972).

[b0850] Trombetta D., Castelli F., Sarpietro M.G. (2005). Mechanisms of antibacterial action of three monoterpenes. Antimicrob. Agents Chemother..

[b0855] Tundis R., Loizzo M.R., Menichini F. (2008). Biological and pharmacological activities of iridoids: recent developments. Mini Rev. Med. Chem..

[b0860] Ultee A., Kets E., Smid E. (1999). Mechanisms of action of carvacrol on the food-borne pathogen Bacillus cereus. Appl. Environ. Microbiol..

[b0865] Ultee A., Slump R., Steging G. (2000). Antimicrobial activity of carvacrol toward Bacillus cereus on rice. J. Food Prot..

[b0870] van der Velden W.J., van Iersel T.M., Blijlevens N.M. (2009). Safety and tolerability of the antimicrobial peptide human lactoferrin 1–11 (hLF1-11). BMC Med..

[b0875] Ventola, C. L., 2015. The antibiotic resistance crisis: part 1: causes and threats. Pharmacy therapeutics. 40, 277. PMC4378521 25859123

[b0880] Walia K., Madhumathi J., Veeraraghavan B. (2019). Establishing antimicrobial resistance surveillance & research network in India: journey so far. Indian J. Med. Res..

[b0885] Walsh S.E., Maillard J.Y., Russell A. (2003). Activity and mechanisms of action of selected biocidal agents on Gram-positive and-negative bacteria. J. Appl. Microbiol..

[b0890] Wang, T.-T., F. P. Nestel, V. Bourdeau, et al., 2004. Cutting edge: 1, 25-dihydroxyvitamin D3 is a direct inducer of antimicrobial peptide gene expression. 173, 2909-2912. 10.4049/jimmunol.173.5.2909 15322146

[b0895] Wang Y.-P., Lei Q.-Y. (2018). Metabolite sensing and signaling in cell metabolism. Signal Trans. Targ. Ther..

[b0900] Warnock D.W. (2007). Trends in the epidemiology of invasive fungal infections. Nippon Ishinkin Gakkai Zasshi.

[b0905] Weber, G., J. D. Heilborn, C. J. CI, et al., 2005. Vitamin D induces the antimicrobial protein hCAP18 in human skin. 124, 1080-1082. 10.1111/j.0022-202X.2005.23687.x 15854055

[b0910] Williams P. (2007). Quorum sensing, communication and cross-kingdom signalling in the bacterial world. Microbiology.

[b0915] Woo, M.-W., H.-J. Nah, S.-S. Choi, et al., 2014. Pikromycin production stimulation through antibiotic down-regulatory gene disruption in Streptomyces venezuelae. 19, 973-977.

[b0920] Xue, J., P. M. Davidson and Q. Zhong, 2013. Thymol nanoemulsified by whey protein-maltodextrin conjugates: the enhanced emulsifying capacity and antilisterial properties in milk by propylene glycol. Journal of agricultural food chemistry. 61, 12720-12726. 10.1021/jf4043437 24328082

[b0925] Yamshchikov, A. V., N. S. Desai, H. M. Blumberg, et al., 2009. Vitamin D for Treatment and Prevention of Infectious Diseases; A Systematic Review of Randomized Controlled Trials. 15, 438-449. 10.4158/EP09101.ORR PMC2855046 19491064

[b0930] Ye L., Zhang J., Xiao W. (2020). Efficacy and mechanism of actions of natural antimicrobial drugs. Pharmacol. Ther..

[b0935] Zavascki A.P., Goldani L.Z., Li J. (2007). Polymyxin B for the treatment of multidrug-resistant pathogens: a critical review. J. Antimicrob. Chemother..

[b0940] Zhang L., Kong Y., Wu D. (2008). Three flavonoids targeting the β-hydroxyacyl-acyl carrier protein dehydratase from Helicobacter pylori: crystal structure characterization with enzymatic inhibition assay. Protein Sci..

[b0945] Zida A., Bamba S., Yacouba A. (2017). Anti-Candida albicans natural products, sources of new antifungal drugs: a review. J. Mycol. Med..

